# Development of an *in vitro* biofilm model for the study of the impact of fluoroquinolones on sewer biofilm microbiota

**DOI:** 10.3389/fmicb.2024.1377047

**Published:** 2024-03-27

**Authors:** Sarah A. Naudin, Aude A. Ferran, Pedro Henrique Imazaki, Nathalie Arpaillange, Camille Marcuzzo, Maïna Vienne, Sofia Demmou, Alain Bousquet-Mélou, Felipe Ramon-Portugal, Marlene Z. Lacroix, Claire Hoede, Maialen Barret, Véronique Dupouy, Delphine Bibbal

**Affiliations:** ^1^INTHERES, Université de Toulouse, INRAE, ENVT, Toulouse, France; ^2^Université de Toulouse, INRAE, BioinfOmics, GenoToul Bioinformatics Facility, Castanet-Tolosan, France; ^3^Université de Toulouse, INRAE, UR 875 MIAT, Castanet-Tolosan, France; ^4^Centre de Recherche sur la Biodiversité et l’Environnement, Université de Toulouse, CNRS, IRD, Toulouse INP, Université de Toulouse, Toulouse, France

**Keywords:** wastewater, sewer, biofilm, fluoroquinolone, bioreactor, antibiotic resistance

## Abstract

Sewer biofilms are likely to constitute hotspots for selecting and accumulating antibiotic-resistant bacteria (ARB) and antibiotic resistance genes (ARGs). This study aimed to optimize culture conditions to obtain *in vitro* biofilms, mimicking the biofilm collected in sewers, to study the impact of fluoroquinolones (FQs) on sewer biofilm microbiota. Biofilms were grown on coupons in CDC Biofilm Reactors®, continuously fed with nutrients and inoculum (1/100 diluted wastewater). Different culture conditions were tested: (i) initial inoculum: diluted wastewater with or without sewer biofilm, (ii) coupon material: concrete vs. polycarbonate, and (iii) time of culture: 7 versus 14 days. This study found that the biomass was highest when *in vitro* biofilms were formed on concrete coupons. The biofilm taxonomic diversity was not affected by adding sewer biofilm to the initial inoculum nor by the coupon material. *Pseudomonadales, Burkholderiales* and *Enterobacterales* dominated in the sewer biofilm composition, whereas *in vitro* biofilms were mainly composed of *Enterobacterales*. The relative abundance of *qnrA, B, D* and *S* genes was higher in *in vitro* biofilms than sewer biofilm. The resistome of sewer biofilm showed the highest Shannon diversity index compared to wastewater and *in vitro* biofilms. A PCoA analysis showed differentiation of samples according to the nature of the sample, and a Procrustes analysis showed that the ARG changes observed were linked to changes in the microbial community. The following growing conditions were selected for *in vitro* biofilms: concrete coupons, initial inoculation with sewer biofilm, and a culture duration of 14 days. Then, biofilms were established under high and low concentrations of FQs to validate our *in vitro* biofilm model. Fluoroquinolone exposure had no significant impact on the abundance of *qnr* genes, but high concentration exposure increased the proportion of mutations in *gyr*A (codons S83L and D87N) and *par*C (codon S80I). In conclusion, this study allowed the determination of the culture conditions to develop an *in vitro* model of sewer biofilm; and was successfully used to investigate the impact of FQs on sewer microbiota. In the future, this setup could be used to clarify the role of sewer biofilms in disseminating resistance to FQs in the environment.

## 1 Introduction

The first global impact analysis of antibiotic resistance estimated that antibiotic resistance caused 1.27 million deaths worldwide in 2019 (Murray et al., 2022). This study revealed the magnitude of antibiotic resistance’s global threat to human health and proposed measures to save lives. Measures to combat antibiotic resistance are not limited to clinical and community settings. This global ecological problem is addressed through a “One Health” approach, which considers the interconnections between the human, animal and environmental spheres (Hernando-Amado et al., 2019).

The environment is a reservoir of antibiotic-resistant bacteria (ARB) and antibiotic resistance genes (ARGs), either naturally occurring or introduced by the discharge of human and animal effluents. The environment is thought to be both a place of emergence and a route of dissemination of antibiotic resistance (Wellington et al., 2013; Bengtsson-Palme et al., 2018). For example, the quinolone resistance gene *qnrA*, commonly found in clinical enterobacteria isolates, would have been acquired by horizontal transfer from the aquatic species *Shewanella algae* (Poirel et al., 2005). In connection with human activities, the environment is also contaminated by a mixture of chemical agents, including antibiotics, biocides and heavy metals, which can exert selection pressure, enriching the antibiotic resistance reservoir (Buelow et al., 2021).

Wastewater treatment plants (WWTPs) are one of the main focal point for the emergence of resistance in pathogens because favorable conditions are found, including the presence of commensal, pathogenic, and environmental bacteria at high concentrations and cocktails of chemical agents, including antibiotics (Rizzo et al., 2013). Apart from effluents from pharmaceutical industries producing antibiotics, antibiotic residues are present at sub-inhibitory concentrations in wastewater due to antibiotic use in human and veterinary medicine (Chow et al., 2021). Several studies have shown that these sub-inhibitory concentrations can lead to antibiotic resistance selection (Andersson and Hughes, 2014; Sanchez-Cid et al., 2022). Precursor studies defined the minimal selective concentration (MSC) as the smallest concentration that allows the selection of the resistant mutant at the expense of the susceptible strain (Gullberg et al., 2011; Liu et al., 2011). Competition curves between a sensitive bacterium and its isogenic resistant mutant must be performed to determine MSCs. This approach has several limitations in the context of complex microbial communities, as it has been shown that the MSC assessed in a semi-natural microbial community (pig fecal flora) is higher than that assessed when the competition curves are performed without this microbial community (Klumper et al., 2019). Other authors have developed an approach to determine the lowest observed effect concentration (LOEC) on complex aquatic bacterial communities using phenotypic (culture-based methods) and genotypic (qPCR and metagenomic analyses) criteria (Lundstrom et al., 2016; Murray et al., 2018). Such studies aim to determine the predicted no-effect concentrations for resistance (PNEC-Rs) to assess the risk of antibiotic resistance selection in the environment (Murray et al., 2021).

Fluoroquinolones (FQs) are frequently detected in wastewater and environmental matrices because these molecules are persistent in the environment (Mathur et al., 2021). In France and the United Kingdom, the comparison of measured environmental FQ concentrations to PNEC-Rs highlighted that these antibiotics represented a high risk of selection of resistant bacteria in treated wastewater (Haenni et al., 2022; Hayes et al., 2022). FQ resistance mechanisms are also detected in bacteria from the environment. For example, plasmid-mediated quinolone resistance (PMQR) genes, particularly *qnr* genes, have been detected and quantified in WWTPs (Pazda et al., 2019). These genes reduce susceptibility, but usually not to the point of clinical non-susceptibility (Hooper and Jacoby, 2016). In *Escherichia coli*, most resistance mutations generating clinical resistance to FQs are located in the quinolone resistance-determining region (QRDR) in *gyrA* and *parC* genes. These resistance mutations have been shown common in *E. coli* isolated from the environment, even in the absence of FQs (Johnning et al., 2015).

In the context of research exploring the relationship between antibiotic exposure and the selection of resistance in the environment, it would be interesting to focus on the effects of FQs on a particular compartment: the biofilms from sewers. Indeed, until recently, only a few studies have been conducted on sewer biofilms. However, the internal walls of the sewers are covered with biofilms composed of very dense microbial communities, continually exposed to cocktails of chemical agents, including antibiotic residues, with the highest concentrations detected at the entries of WWTPs (Jelic et al., 2015). Moreover, these bacterial communities have also been shown to contain many ARGs (Balcázar et al., 2015; Auguet et al., 2017; Flores-Vargas et al., 2021). In this way, sewer biofilms are likely to be hotspots for the selection and accumulation of ARB and ARGs. Additionally, biofilms in aquatic environments have been identified as focal points for horizontal gene transfer (HGT) of ARGs (Abe et al., 2020).

This study aimed to develop an *in vitro* biofilm model for studying the impact of FQs on bacterial communities in sewer biofilms. Different culture conditions were tested in bioreactors to determine their effect on *in vitro* biofilm production. Once the culture conditions were set, *in vitro* biofilms were exposed to FQs at high and low concentrations, and changes in taxonomic composition, abundances of PMQR genes and proportions of mutations in *gyrA* and *parC* were tracked.

## 2 Materials and methods

### 2.1 Field sample collection and processing

Wastewater and sewer biofilm samples were taken at one of the entries of a WWTP, servicing a population equivalent to 300,000 inhabitants and including hospital wastewater. Wastewater was sampled during four sampling campaigns (A, B, E, and F) in 2021 (Table 1). An average 24-h sample was performed using automatic samplers. Sewer biofilm was only sampled during the first campaign due to technical constraints. The samples were transported at 4°C to the laboratory and placed at 4°C until processing. Wastewater samples were first filtered (170 μm) to remove the larger particles, and used to inoculate bioreactors, as described below. Aliquots of the filtered wastewater were stored in plastic tubes at −80° C until FQ quantification, and others were concentrated 100 times by centrifugation (6,000 ×*g*, 10 min), and were stored at −80°C until DNA extraction. Sewer biofilm was homogenized with 0.9% saline in a homogenizer-mixer (BagMixer 400 W, Intersciences, France), subjected to sonication for 15 min, and filtered using the BagMixer (280 μm). The filtrate was centrifuged for 10 min at 6,000 ×*g*, and the pellets were resuspended in 30 mL of peptone glycerol water (both from Sigma-Aldrich, United States) at 30%. Aliquots of resuspended pellets were stored at −80°C until use for the initial inoculation of bioreactors. Aliquots of sewer biofilm were stored at −80°C for further analysis, as described below.

**Table 1:** Date of wastewater sampling campaigns, counts of heterotrophic bacteria and *Escherichia coli* and percentage of resistance to ciprofloxacin.

Sampling campaign ID	Sampling date	Heterotrophic bacteria counts (log_10_ CFU/mL)	Heterotrophic bacteria resistant to ciprofloxacin (%)	*E. coli* counts (log_10_ CFU/mL)	*E. coli* resistant to ciprofloxacin (%)
A	03/02/2021	6.5	7.7	4.4	5.7
B	03/23/2021	5.9	6.8	3.0	7.0
E	06/08/2021	7.6	0.2	4.2	2.6
F	07/13/2021	6.9	3.6	3.8	1.8
Mean (SD)		6.7 (0.7)	4.6 (3.4)	3.8 (0.6)	4.3 (2.5)

### 2.2 Bioreactor experiments

The sewer environment was simulated using CDC Biofilm Reactors® (Biosurface Technologies, United States). Bioreactors were fed continuously (0.1 mL/min) with nutrients (R2A broth without magnesium sulfate) and inoculum (1/100 v/v diluted wastewater in R2A broth without magnesium sulfate). The R2A composition for 1 L is as follows: 0.5 g yeast extract (ThermoFisher scientific, United States), 0.5 g proteose-peptone (Sigma-Aldrich, United States), 0.5 g Hy-Case® SF (Sigma-Aldrich, United States), 0.5 g D-(+)-Glucose (Sigma-Aldrich, United States), 0.5 g starch (Acros Organics, United States), 0.3 g potassium phosphate dibasic (Sigma-Aldrich, United States). Wastewater was diluted in order to prevent the impact of chemical agents. Feeding bottles containing nutrients and inoculum were placed at 4°C. At the start of the experiment, the bioreactors were filled with 350 mL of a mixture containing 1/100 diluted wastewater and R2A broth without magnesium sulfate (1:1 v/v). The bioreactors were kept at 21°C. The cultures were stirred on a magnetic plate at 100 rpm. Biofilms were formed on removable coupons placed on rods supported by the top of the bioreactor. Each bioreactor had eight rods containing three coupons each (Supplementary Figure S1).

Four experiment sets were conducted (Supplementary Table S1). The first two experiments aimed to optimize the culture conditions to obtain an *in vitro* biofilm mimicking the biofilm collected in sewers. Wastewater collected at campaigns A and B was used to inoculate the first and the second experiment, respectively. Different culture conditions were tested: (i) initial inoculum: diluted wastewater with vs. without sewer biofilm, (ii) coupon material: concrete (C) vs. polycarbonate (PC), and (iii) time of culture: 7 days (D07) vs. 14 days (D14). In each experiment, four bioreactors were used in parallel; sewer biofilm and diluted wastewater were used for the initial inoculation of two bioreactors, and diluted wastewater alone for the other two. In addition, each bioreactor was equipped with 12 polycarbonate coupons and 12 concrete coupons (Supplementary Figure S1). Once the growth conditions were set (concrete coupons, sewer biofilm in the initial inoculum, and an incubation time of 14 days), the impact of FQs on *in vitro* biofilm microbiota was evaluated in two other experiments.

The third and fourth experiments aimed to evaluate the impact of FQs on bacterial communities in sewer biofilms. The inoculation was performed with wastewater from campaign E (third experiment) and F (fourth experiment). Biofilms were established under high and low concentrations of FQs. In the 3rd experiment, ciprofloxacin (CIP) (≥98% purity, Sigma-Aldrich, United States) and norfloxacin (NOR) (≥98% purity, Sigma-Aldrich, United States) were added at 5,000 μg/L (high exposure), and at 2.5 μg/L (low exposure) in the 4th. At the start of the experiments, FQs were added to the initial inoculum, and bioreactors were fed with R2A broth containing FQs to obtain target concentrations in bioreactors. For each FQ exposure experiment, three conditions were tested: (i) control (no FQ), (ii) CIP, and (iii) NOR (Supplementary Figure S1).

Biofilms formed on coupons were collected on D07 and D14. During the experiments dedicated to the testing of different growing conditions (first and second experiments), at each sampling time, two rods containing concrete coupons (one replicate) and two rods containing polycarbonate coupons (one replicate) were taken from each bioreactor (Supplementary Figure S1). During the FQ exposure experiments (third and fourth experiments), at each sampling time, two replicates, consisting of two rods, were taken from each bioreactor (Supplementary Figure S1). After rinsing with 0.9% saline, the biofilm was harvested by swabbing the external surface of the three coupons of the same rod. The operation was repeated for the coupons of the other rod of the replicate. The two swabs were placed in 0.9% saline and subjected to sonication for 5 min. The sample was concentrated and stored at −80°C prior to DNA extraction. Additionally, during FQ exposure experiments, wastewater samples from the bioreactors were collected on days 2, 7, 9, and 14 to quantify FQs. A sample description is available in Supplementary datasheet S1.

### 2.3 Quantification of FQs in bioreactors

CIP and NOR were quantified by ultra-high-performance liquid chromatography with UV and fluorescence detection (Acquity UPLC, Waters, MA United States). One hundred microliters of the sample were added to 100 μL of internal standard (IS, marbofloxacin 0.5 μg/mL for UV detection and danofloxacin 2 ng/mL for fluorescence detection) diluted in 6.6% trichloroacetic acid. The mixture was vortexed for 2 min at 1,400 rpm and 10°C and centrifuged for 10 min at 20,000 ×*g* and 4°C. The supernatant (10 μL) was injected into an Acquity UHPLC® BEH C_18_ column (2.1 × 50 mm, 1.7 μm) (Waters Inc., Milford, MA, United States) and eluted at 40°C and a flow rate of 0.3 mL/min with a gradient consisting of H_2_O, 0.1% formic acid (FA) and methanol (MeOH). Detection was carried out at 278 nm for UV detection, and 280 nm excitation wavelength and 425 nm emission wavelength for fluorescence detection. The concentration ranges for calibration were 0.5–10 μg/L for the low-exposure experiment and 250 ng/mL – 10,000 μg/L for the high-exposure experiment.

### 2.4 Quantification of FQs in field samples

Ten FQs and quinolones (Qs) were assayed in field samples: five FQs for human use (ciprofloxacin, lomefloxacin, moxifloxacin, norfloxacin, and ofloxacin), as well as five FQs/Qs for veterinary use (danofloxacin, enrofloxacin, marbofloxacin, flumequine, and oxolinic acid). All antibiotics were assayed by ultra-high-performance liquid chromatography coupled to a triple quadrupole mass spectrometer (Nexera LCMS8045, Shimadzu, Japan). Samples (10 μL) were eluted at 0.3 mL/min with a gradient consisting of H_2_O/acetonitrile (ACN) acidified with 0.1% FA and at 40°C onto an Acquity UHPLC® BEH C_18_ column (2.1 × 100 mm, 1.7 μm). FQs and Qs were ionized by electrospray in positive mode (ESI+) and detected using multiple reaction monitoring mode. Multiple reaction monitoring transitions, cone voltage, and collision energies are reported in Supplementary Table S2. Wastewater samples (250 μL) were vortexed for 1 min at 1,000 rpm, centrifuged at 14,000 rpm and 4°C for 20 min, and filtered using 0.45 μm-polytetrafluoroethylene (PTFE) syringe filters. Filtrates (95 μL) were completed by adding 5 μL of ACN acidified with 2% FA and 10 μL of IS (ciprofloxacin-d_8_ at 100 ng/mL). The biofilm was previously lyophilized for 48 h and stored at −20°C until extraction. The lyophilized biofilm (200 mg) was mixed with 25 μL of IS (ciprofloxacin-d_8_ 100 ng/mL) and 1 mL of MeOH acidified with 1% FA. The mixture was ground for 3 min at a frequency of 1/30 Hz with two glass beads (Retsch MM400, Verder Scientific GmbH & Co. KG) and then centrifuged at 20,000 ×*g* at 4°C for 10 min. This process was repeated, and the two supernatants were added and evaporated to dryness under nitrogen. The extract was suspended in 250 μL of H_2_O/ACN (95:5 v/v) acidified with 0.1% FA. The mixture was vortexed for 5 min and filtered using a 0.45 μm-PTFE syringe filter. FQs and Qs concentrations were estimated from a calibration curve at concentrations ranging from 0.05 to 10 ng/mL for both matrices. Each sample was directly assayed in a first injection and was enriched with a mixture containing the 10 FQs and Qs at 5 ng/mL for the biofilm and 1 ng/mL for the wastewater to check for matrix effects. The LOQ was set at 0.05 ng/mL for all FQs and LOD was 0.005 ng/mL.

### 2.5 Bacterial counts

R2A agar (as described in 2.2 with 15 g agar (Sigma-Aldrich, United States)) and *E. coli* ChromoSelect Agar B (Sigma-Aldrich, United States) plates were inoculated to count heterotrophic bacteria and *E. coli*, respectively. CIP-resistant bacteria and CIP-resistant *E. coli* were counted on R2A agar supplemented with 2 mg/L of CIP and on ChromoSelect Agar B plates supplemented with 0.25 mg/L of CIP, respectively. R2A agar plates were incubated for 48 h at 31°C, and ChromoSelect Agar B plates overnight at 45°C. Percentage of resistant heterotrophic bacteria and *E. coli* were then calculated, and statistical significance assessed using ANOVA and Dunnett’s test from DescTools package at a 95% family-wise confidence level in RStudio (Version 2023.6.0).

### 2.6 DNA extraction

DNA was extracted from field samples (wastewater and sewer biofilm) using the FastDNA™ SPIN for Soil Kit (MP Biomedicals, United States), as described by the manufacturer, with the PPS and SEWS-M wash steps repeated twice. DNA was extracted from *in vitro* biofilms using the DNeasy Blood and Tissue kit (QIAGEN, The Netherlands), as described by the manufacturer. The purity and quantity of DNA were analyzed by spectrometry with NanoDrop™ One (ThermoFisher Scientific, United States).

### 2.7 Relative abundance of plasmid-mediated quinolone resistance (PMQR) genes

Six plasmid-mediated quinolone resistance (PMQR) genes (*qnrA, qnrB, qnrC, qnrD, qnrS* and *qepA*) and the 16S rRNA gene were quantified. DNA fragments of each target DNA were synthesized and subsequently inserted into pEX-A258 to construct recombinant plasmids (Eurofins Genomics, Luxembourg) and generate standard curves (Supplementary Table S3). DNA extracts were submitted to 35 PCR cycles using the SsoAdvanced Universal SYBR Green Supermix (Bio-Rad, Canada) and a mix of primers (300 nM final concentration each). The cycling conditions were as follows: initial denaturation at 98°C for 3 min, followed by 35 cycles of 98°C for 15 s, 60°C for 15 to 60 s (depending on the targeted gene). Primers for each targeted DNA are listed in Supplementary Table S3. The quantity of each gene was calculated using a standard curve from 10 to 10^8^ copies/μL with Bio-Rad CFX Manager 3.1 analysis software. The number of copies of PMQR genes per μL was normalized by the number of copies of the 16S rRNA gene per μL to assess the relative abundance of PMQR genes in samples. Comparisons between samples were done in RStudio (Version 2023.6.0) and statistical analysis was performed using Kruskal–Wallis test followed by Dunn’s test from FSA package, with Holm’s correction for multiple comparisons, for sample comparison of 1st and 2nd experiments, and Dunnett’s test from DescTools package at the 95% family-wise confidence level for 3rd and 4th experiments.

### 2.8 *Escherichia coli gyrA* and *parC* genes sequencing and analysis

From DNA extracted from selected samples, *gyrA* and *parC* amplicons were obtained from two independent PCRs using AmpliTaqGold360 Master Mix (ThermoFisher Scientific) and a mix of primers (1 μM final concentration each). The cycling conditions consisted of denaturation at 95°C for 6 min, followed by 40 cycles of 95°C for 30 s, 55°C for 45 s, and 72°C for 1 min, followed by 72°C for 7 min. Primers for each targeted DNA are listed in Supplementary Table S3. Resulting PCR products were purified and sequenced at the GeT-Biopuces core facility (Toulouse, France) using Illumina MiSeq V3, yielding 2 × 250 reads leading to 504,000–616,000 sequences per sample for *gyrA* amplicon (311 bp) and 476,000–655,000 sequences per sample for *parC* amplicon (287 bp) per sample. Adapters and low-quality bases were trimmed (−q25 length 150) using TrimGalore (Version 0.6.5). The remaining reads were aligned using bwa-mem (Version 2.2) to a database of *gyrA* and *parC* genes of *E. coli* and *Shigella* (NCBI nucleotide “(*e_coli*[orgn] OR *shigella*[orgn]) AND (*gyrA*[gene] OR *parC*[gene]) AND (*gyrA*[title] OR *parC*[title])”) to select *E. coli* or *Shigella gyrA/parC* reads for subsequent analysis. The paired reads were merged using vsearch - fastq_mergepairs (Version 2.6.2) and dereplicated using u-search - fastx_uniques (Version 11.0.667). Chimeric sequences were removed using usearch - uchime_denovo. After these steps, 2,239–29,271 reads were left for the subsequent analysis for *gyrA* and 13,084 to 42,800 for *parC*. To identify mutations and their abundances in *gyrA/parC*, sequences were aligned read by read to the reference (*E. coli* K-12 MG1655 RefSeq locus tags *gyrA*: b2231 *parC*: b3019) using MAFFT -keeplenght -addfragments (restrict the length of the alignments to the length of the reference sequence).

### 2.9 16S rRNA sequencing and analysis

The V3-V4 region of the 16S rRNA gene was amplified with the primers F343 (CTTTCCCTACACGACGCTCTTCCGATCTACGGRAGGCAGCAG) and R784 (GGAGTTCAGACGTGTGCTCTTCCGATCTTACCAGGGTATCTAATCCT) using 30 amplification cycles with an annealing temperature of 65°C (Drouilhet et al., 2016). Single multiplexing was performed using an in-house 6 bp index, which was added to R784 during a second PCR with 12 cycles using forward primer (AATGATACGGCGACCACCGAGATCTACACTCTTTCCCTACACGAC) and reverse primer (CAAGCAGAAGACGGCATACGAGAT-index-GTGACTGGAGTTCAGACGTGT). The resulting PCR products were purified and loaded onto the Illumina MiSeq cartridge (2 × 250 PE) according to the manufacturer’s instructions. The sequences were analyzed using the standardized pipeline FROGS version 4.1.0 (Escudie et al., 2018). Paired-end reads were merged using Vsearch software (Version 2.17.0) with a 0.1 mismatch rate. Unmerged reads were discarded, and only the amplicons between 400 and 490 bp were kept. Primers were trimmed from the sequences. Merged reads were clustered using the swarm tool (Mahé et al., 2014) with an aggregation distance of 1, and chimeras were removed. Then, clusters containing <0.005% of the total sequences were further filtered as recommended elsewhere (Bokulich et al., 2013). Taxonomic affiliation was completed by blasting the sequences against the 16S SILVA Pintail100 38.1 database (Quast et al., 2013). These parameters were validated with microbial community standards (ZymoBIOMICS, D6300, and D6310), extracted and sequenced with our samples. The resulting data were then computed in RStudio (Version 2023.6.0). The vegan and phyloseq packages were used to analyze alpha and beta diversity. The differences between Shannon diversity were assessed with ANOVA and Tukey’s post-hoc test, with a 95% family-wise confidence level. Constrained analysis of principal components (CAP) and principal coordinate analysis (PCoA) were performed using phyloseq package, and permutational multivariate analysis of variance (PERMANOVA) using the vegan package. PCoA and PERMANOVA were performed using the Bray–Curtis Dissimilarity metric.

### 2.10 Metagenomic analysis

Selected samples were sequenced at the Get-PlaGe core facility (Toulouse, France) using Illumina Novaseq 6000 (2 × 150 bps). They included the sewer biofilm with ~167 million read pairs, four wastewater samples with ~232 million reads pairs, ~190 million reads pairs, ~228 million reads pairs, and ~ 249 Million read pairs, and eight *in vitro* biofilm samples (~169, ~278, ~205, ~225, ~240, ~200, ~208, and ~ 180 million reads pairs). Reads were cleaned using the metagWGS v2.2 workflow cleaning step, meaning adapters were removed and reads trimmed if quality <20 by cutadapt v2.10 and Sickle v1.33. Read pairs mapping the humanGrch38 genome were removed (bwa-mem2 v0.7.17-r1188, samtools v1.10) (Fourquet et al., 2022).^1^ Due to lack of enough sequencing efforts and higher diversity, field samples (sewer biofilm and wastewater) assemblies were much more fragmented than the *in vitro* ones. This difference in diversity between field and *in vitro* samples was highlighted by looking at the histograms of the frequency of kmers (with kat 2.4.2, kmer size of 21). Since our goal was to compare the two in terms of ARG content, we decided not to use the assemblies but the reads so as not to bias the results of the comparison between the two types of samples. These cleaned reads were then used to find and quantify ARGs and efflux pump genes (EPGs) by using RGI v5.2.1 (rgi bwt with bwa aligner, 8 threads, default values for all the other parameters) against CARD database v3.2.1(Alcock et al., 2020). We used sortmerna v4.3.2 (Kopylova et al., 2012) against the 16S database provided here:^2^ with default parameters to quantify reads corresponding to 16S rRNA gene. Then, ARGs/EPGs were filtered according to reads quality (Average % of reference allele(s) covered by reads ≥50, number of reads completely mapped to these alleles ≥10, average MAPQ (Completely Mapped Reads) ≥ 30 for at least one sample). We excluded ARGs/EPGs for which resistance resulted from point mutational alterations that were species-specific, genes of efflux pumps not known to expel antibiotics, genes conferring resistance by their absence or under-expression, and regulators of efflux pumps (Supplementary datasheet S2). A total of 349 ARGs (Supplementary datasheet S3) and 149 EPGs (Supplementary datasheet S4) were kept. Data were analyzed in R (version 4.2.2) using phyloseq package (version 1.42.0). To account for possible variations in bacterial proportion reads across samples, we normalized by the 16S rRNA reads. We created a matrix for ARG or EPG counts that included the 16S rRNA counts and resampled it with the lowest 16S rRNA count among libraries (126,759 reads in the sewer biofilm). This was achieved using rarefy function from R package “phyloseq”. Thus, normalized 16S reads were similar to those of the sewer biofilm sample, and the proportion of ARG/16S rRNA or EPGs/16S rRNA is maintained in each of the samples after normalization. We then calculated the alpha and beta diversity. Wilcoxon rank-sum (Mann–Whitney) test was used to compare Shannon diversity between *in vitro* biofilm and wastewater. PCoA was performed using the Bray–Curtis Dissimilarity metric. The differential abundance of ARGs/EPGs reads between *in vitro* biofilm and another sample type (sewer biofilm or wastewater) was visualized with the Log2 fold change ratio. To limit false positive, only genes showing a Log2FC ≥ 3.32 or ≤ −3.32 (representing a change of at least 10-fold) were taken into account when at least one of the 2 types of samples contained a minimum of 100 reads (after rarefaction). To compare *in vitro* biofilm to wastewater, the p-adjusted value obtained from poisson linear model and a FDR correction for multiple testing were applied.

### 2.11 Procrustes analysis

Procrustes analysis was performed using the R vegan package to determine whether the changes in ARGs revealed by metagenomic were linked to microbial community changes showed by 16S rRNA sequencing. ARGs data from metagenomics were rarefied to the lowest 16S rRNA. Corresponding 16S rRNA sequencing samples were reanalyzed as described previously. PCoA was performed using the Bray-Curtis distance for both datasets, and similarity was assessed using the “protest” function with 99,999 permutations.

## 3 Results

### 3.1 Fluoroquinolones and ciprofloxacin resistant bacteria in field samples

Among the ten Qs/FQs analyzed in wastewater and biofilms, only ciprofloxacin and ofloxacin were detected in wastewater at 187.5 ± 63.3 and 363.2 ± 97.6 ng/L, respectively. In sewer biofilm, ciprofloxacin, norfloxacin and ofloxacin concentrations were 3,900, 1,330, and 48,410 ng/kg of dry matter, respectively (Table 2). The concentration of the other antibiotics remained below the LOD.

**Table 2:** Fluoroquinolone concentration in field samples (wastewater and biofilm) and bioreactors during FQ exposure experiments.

FQ concentration			
	CIP (ng/L)	NOR (ng/L)	OFL (ng/L)
*Wastewater (campaign ID)*			
A	182.9	<LOD	408.2
B	122.8	<LOD	240.9
E	274.2	<LOD	467.5
F	170.0	<LOD	336.2
Mean (SD)	187.5 (63.3)	<LOD	363.2 (97.6)
	**CIP (ng/kg of dry matter)**	**NOR (ng/kg of dry matter)**	**OFL (ng/kg of dry matter)**
Sewer biofilm	3,900	1,330	48,410
	**CIP (μg/L)**	**NOR (μg/L)**	
**Bioreactors**			
*High-exposure*			
Mean (SD)	2,125.4 (1,749.3)	2,083.9 (1,464.4)	
*Low-exposure*			
Mean (SD)	2.7 (0.2)	2.7 (0.1)	

CIP, ciprofloxacin; NOR, norfloxacin; OFL, ofloxacin; LOD, limit of detection; FQ, fluoroquinolone.

Bacteria counts in wastewater ranged from 5.9 to 7.6 log_10_ CFU/mL for heterotrophic bacteria, and from 3.0 to 4.4 log_10_ CFU/mL for *E. coli* (Table 1). The mean of CIP-resistant heterotrophic bacteria and *E. coli* in the wastewater of the four campaigns was 4.6 ± 3.4% and 4.3 ± 2.5%, respectively. In sewer biofilm, these percentages were 6.0 and 2.4% for CIP-resistant heterotrophic bacteria and *E. coli*, respectively.

### 3.2 Optimization of *in vitro* biofilm culture conditions

#### 3.2.1 Bacterial biomass

The results of counts of heterotrophic bacteria in *in vitro* biofilms showed that there was no significant difference in bacterial counts between biofilms obtained following the initial inoculation of the bioreactors with wastewater, whether or not supplemented with sewer biofilm (Kruskal–Wallis, *p* = 0.598). Similarly, no difference was observed between biofilms harvested after 7 or 14 days of culture (Kruskal–Wallis, *p* = 0.365; Supplementary Figure S2A). On the other hand, a significant difference was observed between the bacterial counts of biofilms formed on concrete and polycarbonate coupons. Indeed, concrete coupons facilitated a higher level of bacteria forming the biofilm (Kruskal–Wallis, *p* = 2.31 × 10^−5^). The quantification of 16S rRNA, as a marker of bacterial biomass, also demonstrated significantly greater quantities of biomass for the biofilms formed on concrete coupons (Supplementary Figure S2C). Regarding *E. coli* counts, the effect of coupon material on biofilm formation was not significant (Supplementary Figure S2B).

#### 3.2.2 Taxonomic composition

**Figure 1:**
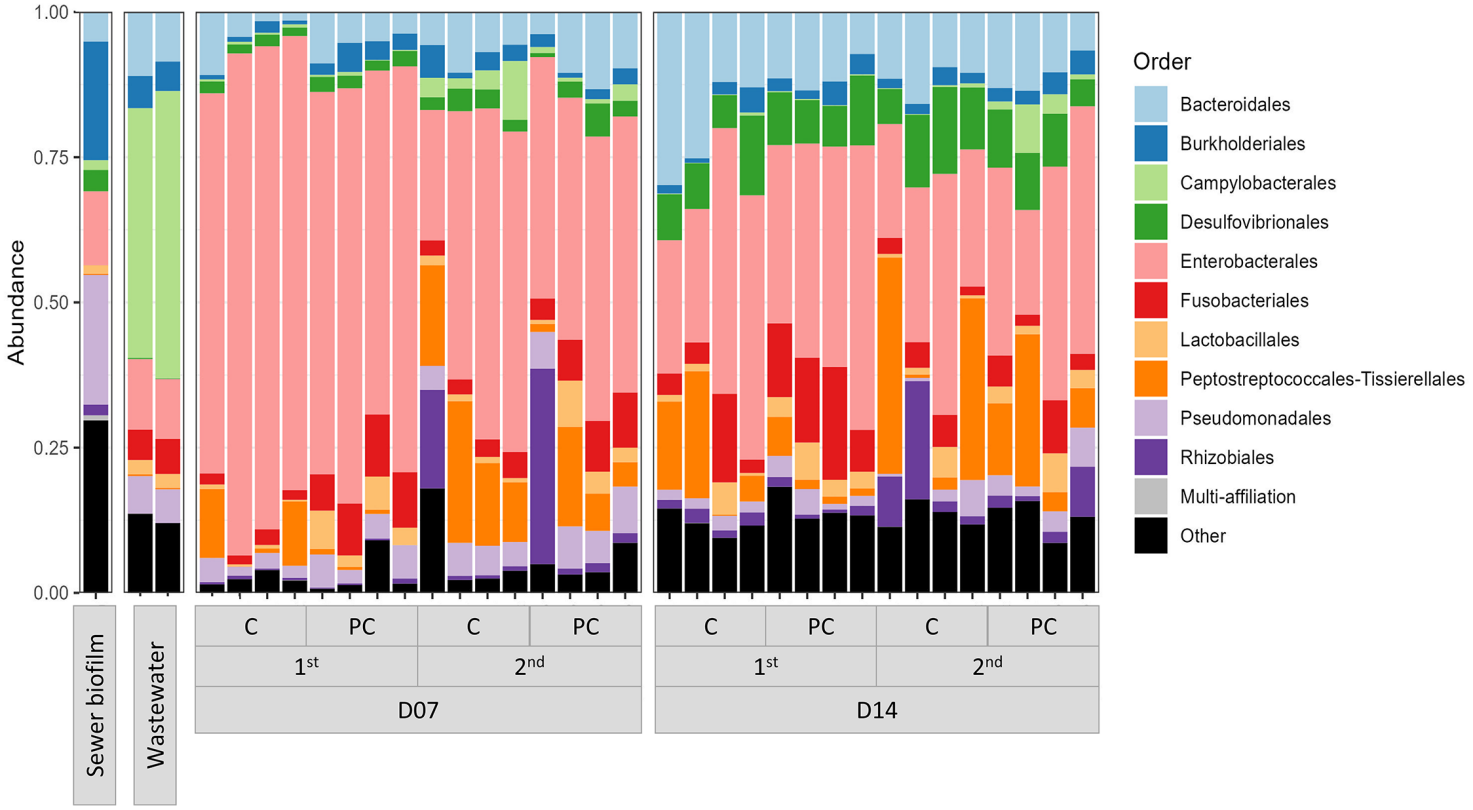
Bar chart representing the relative abundance of the ten most abundant orders in field samples (wastewater and sewer biofilm) and *in vitro* biofilms. The samples were categorized based on sampling time (D07 and D14), experiment set (first and second), and coupon material (C, concrete; PC, polycarbonate).

**Figure 2:**
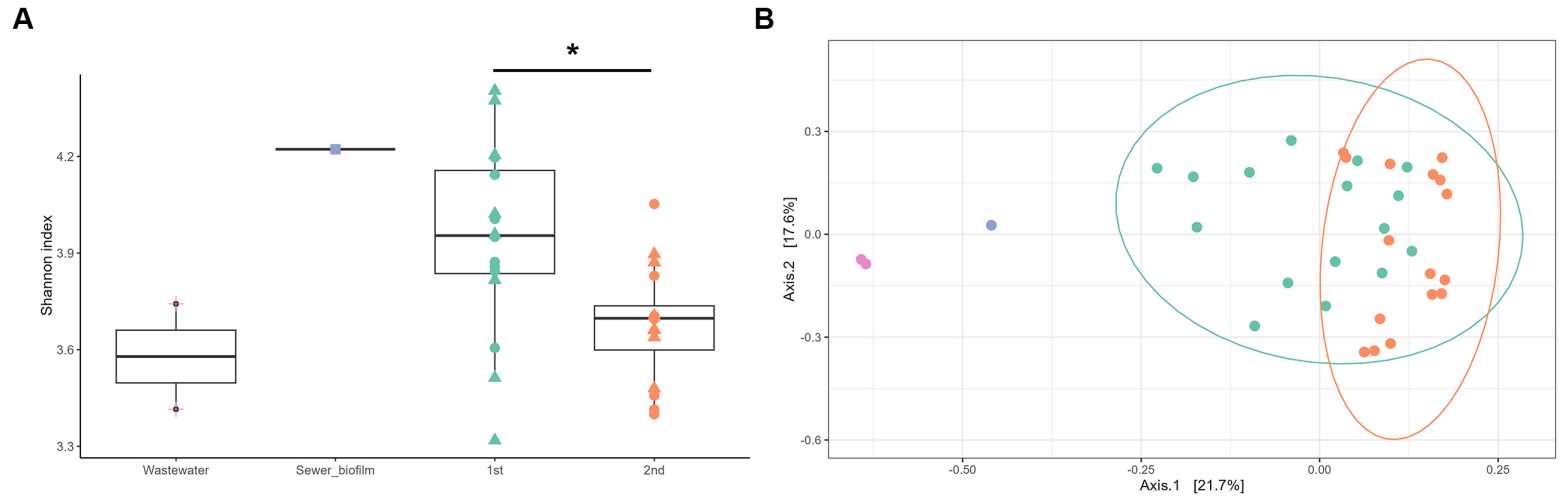
**(A)** Alpha diversity (Shannon index) of field samples (wastewater, purple and sewer biofilm, blue) and *in vitro* biofilms as a function of the experiment set (first, green and second, orange) and the day of sampling (D_7_, circle, and D_14_, triangle). Statistical differences were assessed with ANOVA (*p* < 0.01) and Tukey *post-hoc* test (^*^p < 0.05). **(B)** Visualization by principal coordinate analysis (PCoA) of the beta-diversity analysis carried out using the Bray-Curtis distance, constrained by the type of samples (wastewater, purple; sewer biofilm, blue; first set of experiment, green and second set of experiment, orange).

The taxonomic composition determined by sequencing 16S rRNA V3-V4 section from amplicon as described in section 2.9 showed that *in vitro* biofilms were predominantly composed of *Enterobacterales* (42.8% on average) (Figure 1). The impact of three parameters on microbial diversity was assessed: (i) the material of coupons (concrete vs. polycarbonate), (ii) the initial inoculation (wastewater supplemented or not with sewer biofilm), and (iii) the duration of culture (7 vs. 14 days). These factors did not exhibit any effect on alpha diversity, as estimated by the Shannon index [ANOVA *p* = 0.251 (i), *p* = 0.115 (ii) and *p* = 0.288 (iii)]. However, differences in alpha diversity (Shannon index) based on the experiment set (first vs. second experiment) were observed (ANOVA *p* = 0.008; Figure 2A). A dissimilarity matrix using the Bray-Curtis distance was calculated to compare the bacterial compositions of *in vitro* biofilms considering the experiment set and the sampling day. The CAP plot revealed that communities were primarily differentiated by the sampling day, with also an influence from the experiment set (Supplementary Figure S3).

Moreover, considering field samples, a PCoA analysis on the Bray-Curtis distance showed a separation of samples according to their type (wastewater, sewer biofilm, and *in vitro* biofilms) (Figure 2B). It also revealed a distinction between *in vitro* biofilms from the first and second experiment sets. The relative abundance of the ten most prevalent orders in field samples (wastewater and sewer biofilm) and *in vitro* biofilms showed that samples share common orders but in varying proportions (Figure 1). Sewer biofilm was primarily characterized by *Burkholderiales* (21.8%), *Pseudomonadales* (12.6%) and *Enterobacterales* (11.2%). Wastewater samples were dominated by *Campylobacterales* (39.9% on average). After rarefaction, the heatmap of the most abundant genera indicated that *Pseudomonas* was the predominant genus in sewer biofilm, while *Arcobacter* dominated wastewater (Supplementary Figure S4). The most abundant genera in *in vitro* biofilms were scarce in sewer biofilm.

#### 3.2.3 Resistance to FQs

**Figure 3:**
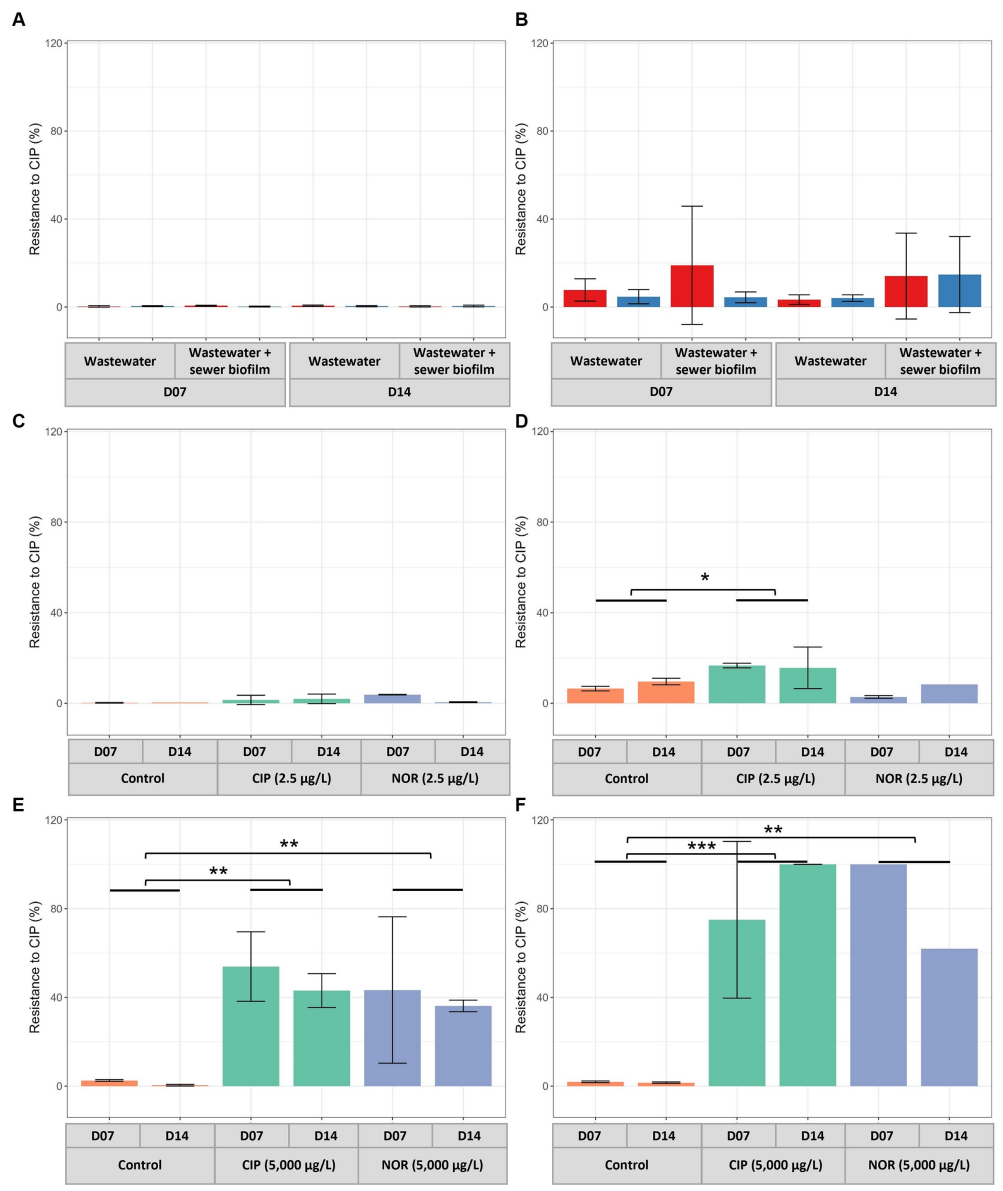
Percentages of resistance to ciprofloxacin of heterotrophic bacteria **(A,C,E)** and *E. coli* **(B,D,F)** in *in vitro* biofilms at D07 and D14. Experiments were conducted without exposure to FQs **(A,B)** with two coupon materials (concrete, red and polycarbonate, blue) and with exposure to FQs at low concentration (2.5 μg/L) **(C,D)** and high concentration (5,000 μg/L) **(E,F)**. CIP, ciprofloxacin; NOR, norfloxacin. *p*-values for **C-F** were calculated with Dunnett’s multiple comparison test (^*^p < 0.05, ^**^p < 0.01, ^***^p < 0.001).

**Figure 4:**
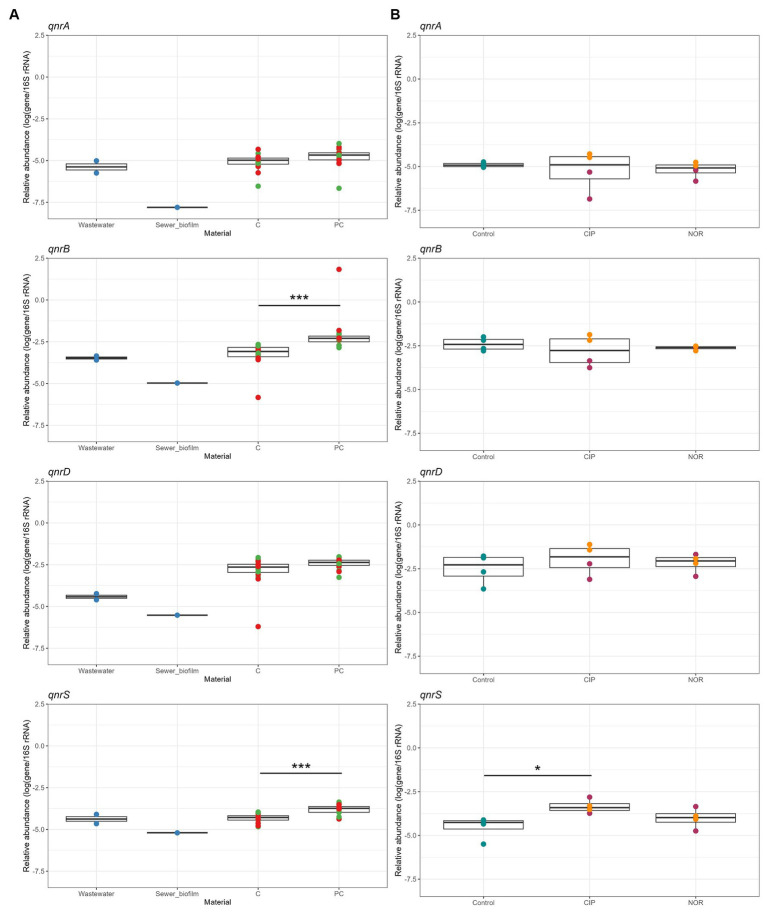
**(A)** Relative abundance of *qnr* genes in field samples (wastewater and sewer biofilm, blue) and *in vitro* biofilms from the 1st and 2nd experiment at D07 and D14, depending on the addition (red) or not (green) of sewer biofilm at the initial inoculation of bioreactors, and the material of the coupons (C, concrete; PC, polycarbonate). **(B)** Relative abundance of *qnr* genes in *in vitro* biofilms not exposed (Control) and exposed to ciprofloxacin (CIP) and norfloxacin (NOR) at high (5,000 μg/L, purple) and low (2.5 μg/L, orange) concentration. Statistical differences were assessed with Kruskal–Wallis test and Dunn *post-hoc* test for **(A)**, and Dunnett’s multiple comparison test for **(B)** (^*^p < 0.05; ^***^p < 0.001).

The percentages of CIP-resistant heterotrophic bacteria were consistently low in *in vitro* biofilms across the tested conditions (Figure 3A). This pattern was similarly observed for the percentages of CIP-resistant *E. coli* (Figure 3B). Concerning the quantification of PMQR genes, *qnrC* and *qepA* genes were not detected in any sample. A significantly higher relative abundance for biofilms obtained on polycarbonate coupons than concrete coupons was observed for *qnrB* and *qnrS* genes. The relative abundances of *qnrA, qnrB, qnrD* and *qnrS* tended to be higher in *in vitro* biofilms than in sewer biofilms but were similar to those in wastewater (Figure 4A).

Additionally, sequencing of *gyrA* and *parC* amplicons from DNA extracted from both field samples and *in vitro* biofilms from the first experiment at D14 was conducted. The most common non-synonymous mutations in *gyrA* were observed in codon S83 (11.0–31.6%) and D87 (10.1–23.7%) and in *parC* in codon S80 (17.1–28.0%) and E84 (7.9–10.5%) (Table 3). Conditions tested in the formation of *in vitro* biofilms did not impact the relative abundance mutations, and they closely resembled those observed for field samples. The most common substitutions in *gyrA* were S83L and D87N in *in vitro* biofilms (16.0 ± 4.5% and 12.0 ± 5.4%) and in wastewater (14.3 and 7.3%), whereas they were S83G (6.7%) and D87L (6.2%) in sewer biofilm (Supplementary Table S4). The relative abundance of co-occurrence of mutations in *gyrA* at codons S83L and D87N ranged from 0.3% (sewer biofilm) to 16.5% (*in vitro* biofilm) (Table 4). The most common substitution in *parC* was S80I for all samples (Supplementary Table S5).

**Table 3:** Relative abundance of mutations in *Escherichia coli gyrA* (S83 and D87) and *parC* (S80 and E84) in field samples, *in vitro* biofilms at D_14_ not exposed to fluoroquinolones considering the coupon material (polycarbonate and concrete) and the supplementation or not of the initial inoculum of bioreactors with sewer biofilm at the initial inoculation of bioreactors, and *in vitro* biofilms at D_14_ exposed to FQs (CIP, ciprofloxacin and NOR, norfloxacin) at low and high concentrations.

Relative abundance of mutations in *E. coli* (%)							
	Field samples		*In vitro* biofilms without FQs				
	Wastewater	Sewer biofilm	Polycarbonate	Polycarbonate	Concrete	Concrete	Mean (SD)
			Without sewer biofilm	With sewer biofilm	Without sewer biofilm	With sewer biofilm	
*gyrA* S83	24.7	11.0	28.1	19.9	31.6	21.0	25.2 (5.6)
*gyrA* D87	15.4	10.1	20.8	13.3	23.7	14.4	18.1 (5.0)
*parC* S80	17.6	17.1	27.3	17.7	28.0	18.2	22.8 (5.6)
*parC* E84	10.5	9.2	9.7	8.7	9.3	7.9	8.9 (0.8)

			*In vitro* biofilms with FQs					
			Control		CIP		NOR	
					Low-exposure	High-exposure	Low-exposure	High-exposure
*gyrA* S83			16.4	19.3	67.5	95.3	25.8	96.8
*gyrA* D87			15.7	12.9	19.3	97.0	18.4	97.8
*parC* S80			15.7	13.7	27.9	86.0	10.8	86.1
*parC* E84			8.2	8.0	11.3	16.7	10.3	15.5

**Table 4:** Relative abundance of mutations in *Escherichia coli gyrA* (S83L and D87N and co-occurrence of these mutations) and *parC* (S80I) in field samples, *in vitro* biofilms at D14 not exposed to fluoroquinolones considering the coupon material (polycarbonate and concrete) and the supplementation or not of the initial inoculum of bioreactors with sewer biofilm at the initial inoculation of bioreactors, and *in vitro* biofilms at D_14_ exposed to FQs (CIP, ciprofloxacin and NOR, norfloxacin) at low and high concentrations.

Relative abundance of mutations in *E. coli* (%)							
	Field samples		*In vitro* biofilms without FQs				
	Wastewater	Sewer biofilm	Polycarbonate	Polycarbonate	Concrete	Concrete	Mean (SD)
			Without sewer biofilm	With sewer biofilm	Without sewer biofilm	With sewer biofilm	
S83L without D87N	8.8	0.6	6.3	6.7	4.8	6.3	6.0 (0.8)
D87N without S83L	1.8	1.2	1.8	1.8	2.3	2.1	2.0 (0.2)
S83L and D87N	5.5	0.3	11.8	5.2	16.5	6.4	10.0 (5.2)
S80I	8.7	8.3	18.5	9.1	18.9	10.2	14.2 (5.2)

			*In vitro* biofilms with FQs					
			Control		CIP		NOR	
					Low-Exposure	High-exposure	Low-exposure	High-exposure
S83L without D87N			2.9	5.9	51.8	2.2	12.8	2.6
D87N without S83L			1.4	1.7	1.1	7.6	1.6	7.5
S83L and D87N			5.8	3.7	5.5	80.4	5.4	83.0
S80I			9.0	6.0	17.5	75.5	2.4	75.8

#### 3.2.4 Resistome

**Figure 5:**
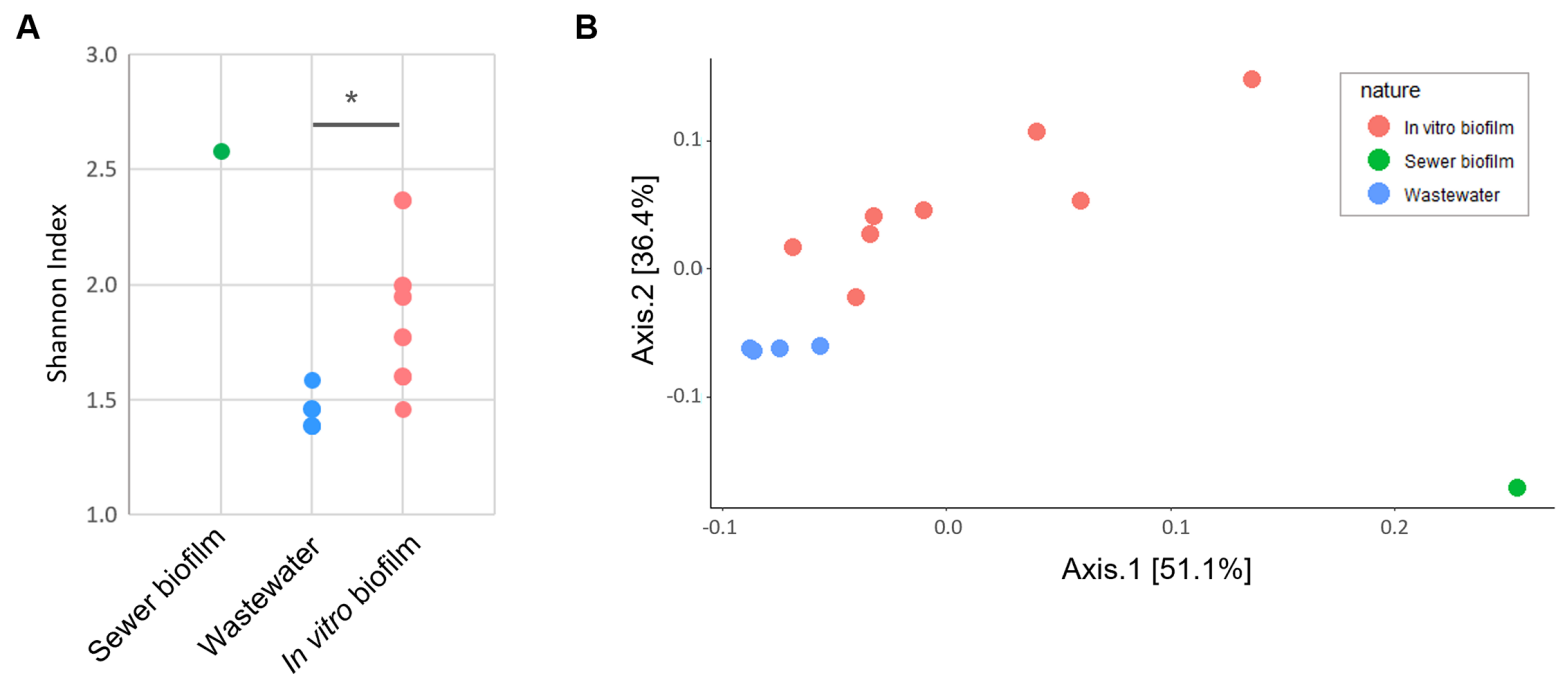
**(A)** Shannon diversity index based on antibiotic resistance genes and efflux pump genes counts derived from metagenomic data (^*^p < 0.05 Mann–Whitney U test). **(B)** PCoA based on Bray-Curtis of ARG and EPG counts for metagenomic data. The ARG and EPG counts were rarified with the lowest 16S rRNA counts.

Thirteen samples, meeting the required quantity and quality parameters, were subjected to metagenomic analysis, including *in vitro* biofilms sampled at D14 on concrete coupons (6 samples) and polycarbonate coupons (2 samples), sewer biofilm (1 sample) and wastewater (4 samples). The sewer biofilm’s resistome (ARGs and EPGs) exhibited the highest Shannon diversity index compared to wastewater and *in vitro* biofilms (Figure 5A). The resistome of *in vitro* biofilms showed a significantly higher Shannon diversity index than that of wastewater (Mann–Whitney, *p* < 0.05). A PCoA analysis on the Bray-Curtis distance illustrated a differentiation of samples based on the nature of the sample (sewer biofilm, wastewater and *in vitro* biofilms) (Figure 5B).

**Figure 6:**
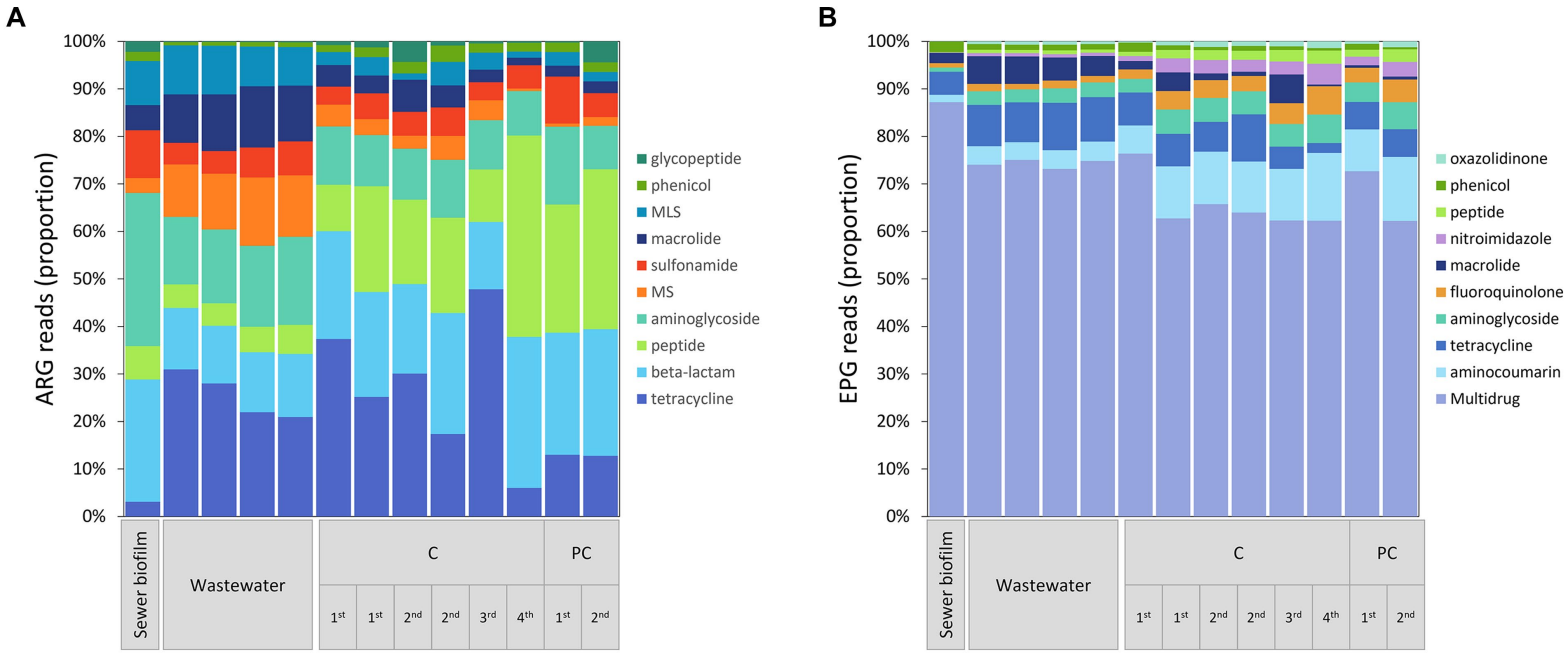
**(A)** Relative abundance of ARGs grouped according to drug family resistance. For MS, and MLS, M, macrolide; L, lincosamide; S, streptogramin. **(B)** Relative-abundance of EPGs grouped according to resistance profile. ARG and EPG that showed an average reads ≥10 in at least one of the sample categories (sewer biofilm, wastewater or *in vitro* biofilm) were included. The samples were grouped by the type of sample, the experiment (1st to 4th) and coupon material (C, concrete; PC, polycarbonate).

For ARG data after rarefaction, 18,139 reads were counted in sewer biofilm, 27,560 ± 528 in wastewater and 20,978 ± 2,008 in *in vitro* biofilms. A total of 349 ARGs were detected and grouped according to the antibiotic classes for which they confer resistance. The results indicated that ARGs conferring resistance to tetracycline and peptides were more abundant in *in vitro* biofilms than in sewer biofilm. Conversely, ARGs conferring resistance to MLS, macrolide or aminoglycoside were less abundant in *in vitro* biofilm than in sewer biofilm (Figure 6A; Supplementary Table S6). Among the 61 ARGs with read counts ≥100 in *in vitro* biofilms or sewer biofilm, 32 were differentially abundant according to the Log2FC estimate: 22 ARGs were under-represented and 10 ARGs were over-represented in *in vitro* biofilm (Supplementary Figure S5). Concerning ARGs conferring resistance to FQs, only three genes (*qnrA1, qnrB35*, and *qnrVC6*) were detected in sewer biofilm, and only the *qnrVC6* contained more than ten reads in one sample (sewer biofilm). Finally, to assess whether the differences in ARGs observed between samples were linked to changes in the microbial community, a Procrustes analysis was performed on the PCoA based on the Bray–Curtis dissimilarity index from metagenomic results and the corresponding 16S rRNA community results. The Procrustes analysis was significant (M^2^ = 0.66, *p* = 0.021).

For EPG data after rarefaction, 92,348 reads were counted in sewer biofilm, 22,162 ± 2,843 in wastewater and 44,081 ± 15,685 in *in vitro* biofilm. Among the 149 EPGs detected, 64 were RND-EP (resistance-nodulation-division efflux pump), primarily involved in multidrug efflux, and 62 were MFS-EP (major facilitator superfamily efflux pump). EPGs were grouped according to the antibiotic classes for which they confer resistance (Figure 6B). The comparison of *in vitro* biofilm to sewer biofilm showed that multidrug, macrolide- and phenicol-specific EPGs were under-represented in *in vitro* biofilm (Figure 6B; Supplementary Table S7). Among the 92 EPGs with read counts ≥100 in *in vitro* biofilms or sewer biofilm, 43 were differentially abundant, 33 being under-represented in *in vitro* biofilm, and ten over-represented in *in vitro* biofilm (Supplementary Figure S6). Regarding resistance to FQs, among the 39 multidrug-EP excluding FQ, 17 were under- and 3 were over-represented in *in vitro* biofilms compared to sewer biofilm. Five FQ-specific EP were detected, including two EPG over-represented in sewer biofilm (*MdtK*, *amrA*) and one under-represented (*ifrA*) compared to sewer biofilm.

### 3.3 FQ exposure experiments

Biofilms were established under two concentrations of FQs: high (5,000 μg/L) and low (2.5 μg/L), with testing of two specific FQs, namely CIP and NOR. The measured FQ exposure concentrations in bioreactors were closely approximated by the nominal target concentrations (Table 2). High exposure to FQs decreased alpha diversity (Shannon index) in the *in vitro* biofilms (Supplementary Figure S7A). Conversely, this reduction in diversity was not observed when biofilms were exposed to low concentrations of FQs. Notably, biofilms formed during the high FQ exposure experiment exhibited a distinct microbial composition compared to other *in vitro* biofilms (Supplementary Figure S7B). The *Peptostreptococcales*-*Tissierellales* order was predominant in all conditions tested during this experiment, including the control ones. It is worth noting that the wastewater used for the inoculation of this experiment set (E) had a microbial composition similar to that of wastewater from the other campaigns (A, B, and F) (Figure 1, Supplementary Figure S7B). The β-diversity between wastewater samples was measured, and campaign E was not specifically more distant (Supplementary Figure S8). High-concentration exposure significantly increased the percentage of CIP-resistant heterotrophic bacteria and *E. coli*, whereas these percentages remained low during low-concentration exposure (Figures 3C–F). Among *qnr* genes, a significantly higher relative abundance was only observed for *qnrS* when exposed to CIP (Figure 4B).

Finally, when *in vitro* biofilms were exposed to low concentrations of FQs, the relative abundance of mutations in *gyrA* S83 ranged from 25.8 to 67.5%, increasing to 95.3–96.8% after high exposure (Table 3). The relative abundance of mutations in *gyrA* D87 remained around 12.9–18.4% in non-exposed or exposed to low concentrations of FQs samples but increased to 97.0–97.8% after high FQ exposure. Reads evidenced S83L and D87N substitutions (Supplementary Table S4). In the absence of FQs and after exposure to low concentrations of FQs, only a few reads containing the S83L substitution showed concomitant D87N substitution (2.4–17.5%), but after high FQ exposition, almost all reads showed both mutations (80.4–83.0%; Table 4). Regarding *parC*, the relative abundance of S80 increased after high FQ exposure (86.0–36.1%), whereas it was not the case for E84 (Table 3). The S80I substitution was predominant (Supplementary Table S5).

## 4 Discussion

This study aimed to establish an *in vitro* biofilm model designed to investigate the influence of FQs on bacterial communities within sewer biofilms. The assessment involved examining the effects of various culture conditions on both the microbial community composition and the characteristics of FQ resistance. Subsequently, upon determining the optimal culture conditions, *in vitro* biofilms were exposed to high and low concentrations of FQs.

### 4.1 Optimization of culture conditions

In the optimization of culture conditions for *in vitro* biofilms cultivation, evaluating three culture parameters was imperative. These parameters involved the coupon material acting as the foundation for subsequent biofilm development, the biofilm development duration, and the composition of the initial inoculum serving as a subtract for biofilm growth. The ensuing discussion analyzes the outcomes of assessing these three parameters, elucidating their impact on the development and characteristics of *in vitro* biofilms.

To explore the potential impact of sewer material on biofilm composition, we conducted tests using two types of coupons designed to support biofilm growth. For instance, recent studies have revealed that the material of pipes can exert a significant influence on both biofilm structure and microbial community within water distribution systems (Lee et al., 2021; Zhang et al., 2022). In our study, concrete coupons were tested because they matched the material used in the pipes at the entrance of the WWTP at which the sewer biofilm had been sampled. Polycarbonate was also tested as it is a material commonly used for the culture of *in vitro* biofilms. Our results showed that using concrete coupons led to biofilms with higher heterotrophic bacteria counts and greater quantities of 16S rRNA compared to polycarbonate coupons. Previous experiments simulating a sewer pipe environment reported similar concentrations of 16S rRNA genes per area for polyvinylchloride (PVC) and concrete (Medina et al., 2020). However, in drinking water distribution systems, biofilms formed on concrete exhibited higher total cell counts and heterotrophic plate counts within 12 days compared to other various materials (copper, stainless steel, cast iron and polyethylene) (Zhu et al., 2014). Finally, the material of the coupons did not impact the taxonomic composition of *in vitro* biofilms, consistent with previous findings that PVC and concrete material did not lead to different prokaryotic communities (Medina et al., 2020). As such, the material of the coupons used in our experiments appears to influence biofilm growth in terms of bacterial biomass but not taxonomic diversity.

In this study, two culture duration were examined to understand how the taxonomic composition of sewer biofilms evolves over time, as highlighted by Zheng et al. (2021), who observed time-dependent bacterial succession in rural sewer biofilms. CAP analysis revealed that microbial community composition varied significantly with incubation time, corroborating Zheng et al.’s findings. This variation likely accounts for the observed differences between the sewer biofilm samples and our *in vitro* biofilms, considering that the latter are relatively younger. A culture duration of 14 days was selected for this reason, as it gave the biofilm more time to establish and get closer in composition to sewer biofilm. Additionally, alpha diversity measures and PCoA analysis indicated distinct microbial communities across different experimental setups. Notably, in one experiment, *in vitro* biofilms formed under high FQ exposure, including control groups, exhibited a different taxonomic composition compared to *in vitro* biofilms from other experiments. While the taxonomic composition of the wastewater used for inoculating the bioreactors appeared similar, subtle variances in these samples could be accentuated under the specific conditions of the bioreactors, leading to variable taxonomic composition, as observed. Even if a single wastewater had been used for the inoculation of all bioreactors, a recent article reported the dominant role of stochastic assembly in creating variations of microbial diversity using a well-controlled laboratory system (Zhou et al., 2013).

Finally, testing the addition of sewer biofilm in the initial inoculation was based on previous findings that sewer biofilms and wastewater share microorganisms, albeit in varying proportions (Auguet et al., 2017; McLellan and Roguet, 2019). The 16S rRNA analysis showed that the dominant microorganisms in the sewer biofilm were from the *Burkholderiales* and *Pseudomonadales* orders, consistent with the review by Li et al. (2019). In contrast, wastewater bacterial communities were predominantly composed of bacteria from the *Campylobacterota* phylum, especially *Arcobacter*, aligning with studies indicating that wastewater microbiota are distinct from human fecal microbiota (Guo et al., 2019; LaMartina et al., 2021). Interestingly, the taxonomic composition of the *in vitro* biofilms differed significantly from both the sewer biofilm and wastewater, being largely dominated by *Enterobacterales*. Moreover, the initial addition of a sewer biofilm at the onset did not alter the taxonomic composition of the biofilms formed. This observation aligns with Medina et al., who reported distinct dominant family taxa in biofilms from simulated sewer experiments compared to actual sewer biofilms (Medina et al., 2020).

Regarding FQ resistance indicators, the percentage of heterotrophic bacteria and *E. coli* resistant to CIP was low both in sewer and *in vitro* biofilms, indicating limited FQ resistance. Kraupner et al. (2018) also assessed *E. coli* resistance to CIP in biofilms developed from treated sewage effluent. Contrary to our results, they reported a high percentage of heterotrophic bacteria resistant to CIP, attributed to the prevalence of intrinsically resistant bacterial species. Further, we quantified quinolone resistance genes *qnrA*, *qnrB*, *qnrD* and *qnrS* in both field samples and in *in vitro* samples. This approach aligns with Pazda et al., who documented these genes in WWTPs (Pazda et al., 2019). Coupons of polycarbonate and concrete material did not lead to a significant difference in accumulation of ARGs, corroborating the findings of Medina et al. (2020) who showed that pipe material did not affect the abundance of ARGs in *in vitro* biofilms. The most common mutations detected were S83L and D87N in the *gyrA* gene and S80I in the *parC* gene. These mutations, also predominantly found in sediment samples (Johnning et al., 2015), are known to confer FQ resistance in *E. coli* (Hooper and Jacoby, 2016). Overall, the various conditions tested in our study did not significantly impact the levels of FQ resistance.

Metagenomic analysis revealed distinct resistomes in each type of sample: sewer biofilm, wastewater, and *in vitro* biofilms. Given the observed differences in taxonomic composition among these sample types, we performed a Procrustes analysis based on the Bray-Curtis distance to investigate the correlation between observed changes in ARG and the microbial community. The significant results from this analysis confirmed that the observed ARG variations were indeed associated with changes in microbial community composition, as it has been described by others (Bengtsson-Palme et al., 2016; Auguet et al., 2017). For instance, genes *mphG* and *aac(3)-IIIc*, which confer resistance to macrolides and aminoglycosides respectively, were under-represented in *in vitro* biofilms compared to sewer biofilm. According to CARD 3.2.4 (Alcock et al., 2023), these genes are more prevalent in bacteria like *Alcaligenes faecalis* of the *Burkholderiales* order or *Pseudomonas aeruginosa* of the *Pseudomonadales* order, both of which are dominant in sewer biofilm. Conversely, several genes related to resistance against antibiotic peptides (*eptA, eptB, MCR.7.1, bacA,* and *pmrF*) associated with *Enterobacterales* (CARD 3.2.4) were over-represented in *in vitro* biofilms. These genes were also more abundant in *in vitro* biofilms than in sewer biofilm.

### 4.2 Impact of FQ exposure

*In vitro* biofilms were exposed to two concentrations of FQs to assess their effects on microbiota composition and indicators quantifying FQ resistance. A high concentration of 5,000 μg/L was chosen to assess the potential of such level in selecting resistance. This concentration is notably higher than the EUCAST epidemiological “cut-off” of CIP for *E. coli* (60 μg/L) and the clinical breakpoint for *Enterobacterales* (500 μg/L) (EUCAST, 2023). Additionally, a low concentration of 2.5 μg/L was tested to simulate environmental exposure levels. This concentration falls in the range of FQ concentrations typically found in wastewater in various high-income countries, reported to be between 0.1 and 9.9 μg/L (Jelic et al., 2015; Auguet et al., 2017; Hayes et al., 2022). In our samples, among the 10 FQs/Qs screened in wastewater, only CIP and OFL were detected, with average concentrations of 170.0 (±56.1) and 347.1 (±80.4) ng/L respectively, aligning with levels found in treated urban wastewater in France (Haenni et al., 2022). Importantly, the 2.5 μg/L concentration was likely to have an impact on biofilm microbiota, as the PNEC-R of CIP is estimated to be between 0.004 and 10.8 μg/L, depending on the method used for determining the selective concentration of this antibiotic (Hayes et al., 2022).

High FQ concentration exposure resulted in a reduction in biofilm diversity, a phenomenon paralleling the decreased diversity in the digestive microbiota of patients undergoing antibiotic treatment, as reported by Schwartz et al. (2020). Specifically, high FQ concentrations significantly increased the proportion of CIP-resistant heterotrophic bacteria and *E. coli*. Notably, this condition also dramatically increased the relative abundance of chromosomal mutations in key *E. coli* genes: *gyrA* S83 and D87, and *parC* S80. The most frequent substitutions were a co-occurrence of S83L, D87N and S80I, with S83L and D87 almost systematically being detected on the same read. In contrast, at the lower FQ concentration, only the *gyrA* gene showed a significant increase in its relative abundance, and the S83L substitution without the D87N substitution was the most frequent. Furthermore, the relative abundance of *qnrB*, *qnrS*, *qnrD*, and *qnrA* genes remained largely unchanged after FQ exposure, excepted for an increase of *qnrA* gene with the addition of CIP. These observations are consistent with the results of Kraupner et al. (2018), who determined the selective concentration for CIP in complex aquatic biofilms. They reported that a concentration of 10 μg/L drastically increased the percentage of CIP-resistant *E. coli* and selected chromosomal resistance mutations, predominantly the triple mutation in *gyrA* S83L/D87N and *parC* S80I, similar to our findings using amplicon sequencing from community DNA. However, in contrast to the findings of Kraupner et al., who observed an increase in *qnrB*, *qnrD*, and *qnrS* genes at 10 μg/L, our study did not result in the selection of PMQR genes under high exposure conditions. This disparity could be attributed to the fact that PMQR genes typically confer low-level resistance (Hooper and Jacoby, 2016).

## 5 Conclusion

In this study, we optimized an *in vitro* model to investigate the impact of FQs on the microbiota of sewer biofilms. Our experimental design included: (i) the use of concrete coupons, chosen for their capacity to yield higher bacterial biomass; (ii) a 14-day incubation time, facilitating the development of more mature biofilms; and (iii) the addition of sewer biofilm to the initial inoculum. Although the inclusion of sewer biofilm did not significantly influence the experimental outcomes, we maintained this approach to better mimic real-world conditions, with biofilm that can break away and migrate in the sewers, and to potentially incorporate population unique to sewer biofilms. Our results indicated differences between *in vitro* and sewer biofilm in terms of microbial composition, abundance of *qnr* genes, and resistome. This finding aligns with recent research by Buelow et al. (2023), which also reported challenges in reproducing field biofilms *in vitro*, noting particularly that the microbiota and extracellular polymeric substance composition of *in vitro* biofilms were more akin to each other than to their natural counterparts (Buelow et al., 2023). We acknowledge that, like all experimental models, ours may have limitations in accurately representing the resistome and microbiota of actual sewer biofilms. However, we demonstrated that our model was effective in tracking multiple changes in biofilms upon FQ exposure. It had no significant impact on the abundance of *qnr* genes whatever the concentration, but high-concentration exposure increased the proportion of mutations in *gyr*A (codons S83L and D87N) and *par*C (codon S80I). These results underscore the utility of our experimental approach to clarify the role of bacterial communities of sewer biofilms in the dissemination of resistance to FQs in the environment.

## Data availability statement

The datasets presented in this study can be found in online repositories. The names of the repository/repositories and accession number(s) can be found at: https://www.ebi.ac.uk/ena, PRJEB69678.

## Author contributions

SN: Data curation, Formal analysis, Validation, Writing-original draft, Writing-review & editing. AF: Conceptualization, Investigation, Methodology, Writing-review & editing. PI: Conceptualization, Data curation, Formal analysis, Investigation, Methodology, Validation, Writing-review & editing. NA: Investigation, Writing – review & editing. CM: Data curation, Formal analysis, Investigation, Validation, Writing – review & editing. MV: Data curation, Formal analysis, Investigation, Validation, Writing – review & editing. SD: Data curation, Formal analysis, Investigation, Validation, Writing – review & editing. AB-M: Conceptualization, Methodology, Writing-review & editing. FR-P: Conceptualization, Investigation, Methodology, Writing-review & editing. ML: Conceptualization, Investigation, Methodology, Writing-review & editing. CH: Conceptualization, Data curation, Formal analysis, Investigation, Methodology, Validation, Writing-review & editing. MB: Conceptualization, Data curation, Formal analysis, Investigation, Methodology, Validation, Writing-review & editing. VD: Conceptualization, Data curation, Formal analysis, Methodology, Investigation, Validation, Writing-review & editing. DB: Conceptualization, Data curation, Formal analysis, Funding acquisition, Methodology, Investigation, Validation, Writing-original draft, Writing-review & editing.

## Footnotes

^1^ https://forgemia.inra.fr/genotoul-bioinfo/metagwgs

^2^ https://github.com/biocore/sortmerna/tree/master/data/rRNA_databases/silva-bac-16s-id90.fasta
