# Supplementary material for: Development of an in vitro biofilm model for the study of the impact of fluoroquinolones on sewer biofilm microbiota
Source: Front Microbiol. 2024 Mar 27;15:1377047. doi: 10.3389/fmicb.2024.1377047 (PMC11004435; doi:10.3389/fmicb.2024.1377047)
Supplement: Supplementary file 4 [file Data_Sheet_5.docx]

**Development of an *in vitro* biofilm model for the study of the impact of fluoroquinolones on sewer biofilm microbiota**

**Sarah A. Naudin^1^, Aude A. Ferran** **^1^, Pedro Henrique Imazaki^1^, Nathalie Arpaillange^1^, Camille Marcuzzo^1^, Maïna Vienne^2, 3^, Sofia Demmou^4^, Alain Bousquet-Mélou ^1^, Felipe Ramon-Portugal^1^, Marlene Z. Lacroix^1^, Claire Hoede^2, 3^, Maialen Barret^4^, Véronique Dupouy^1^ and Delphine Bibbal^1*^**

^1^INTHERES, Université de Toulouse, INRAE, ENVT, Toulouse, France

^2^Université de Toulouse, INRAE, BioinfOmics, GenoToul Bioinformatics facility, Castanet-Tolosan, France

^3^Université de Toulouse, INRAE, UR 875 MIAT, Castanet-Tolosan, France

^4^Laboratoire Ecologie Fonctionnelle et Environnement, Université de Toulouse, CNRS, Toulouse, France

Supplementary Material


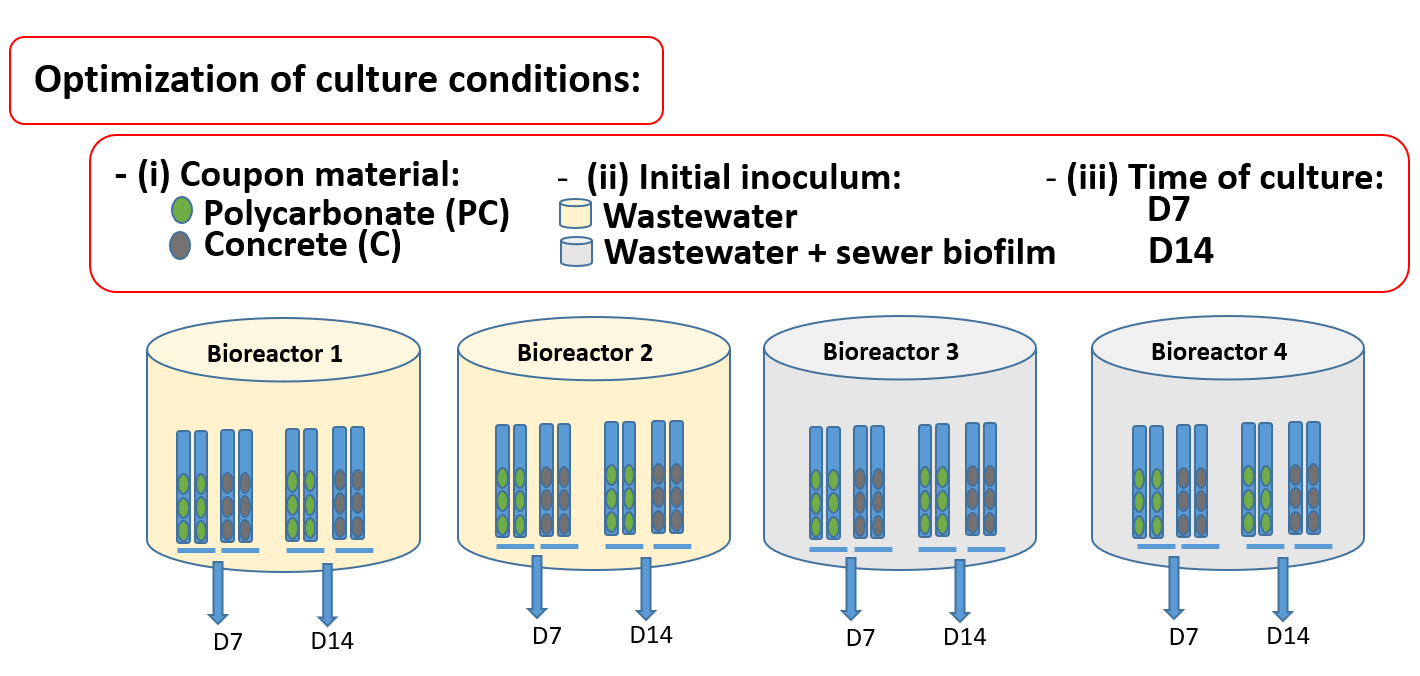

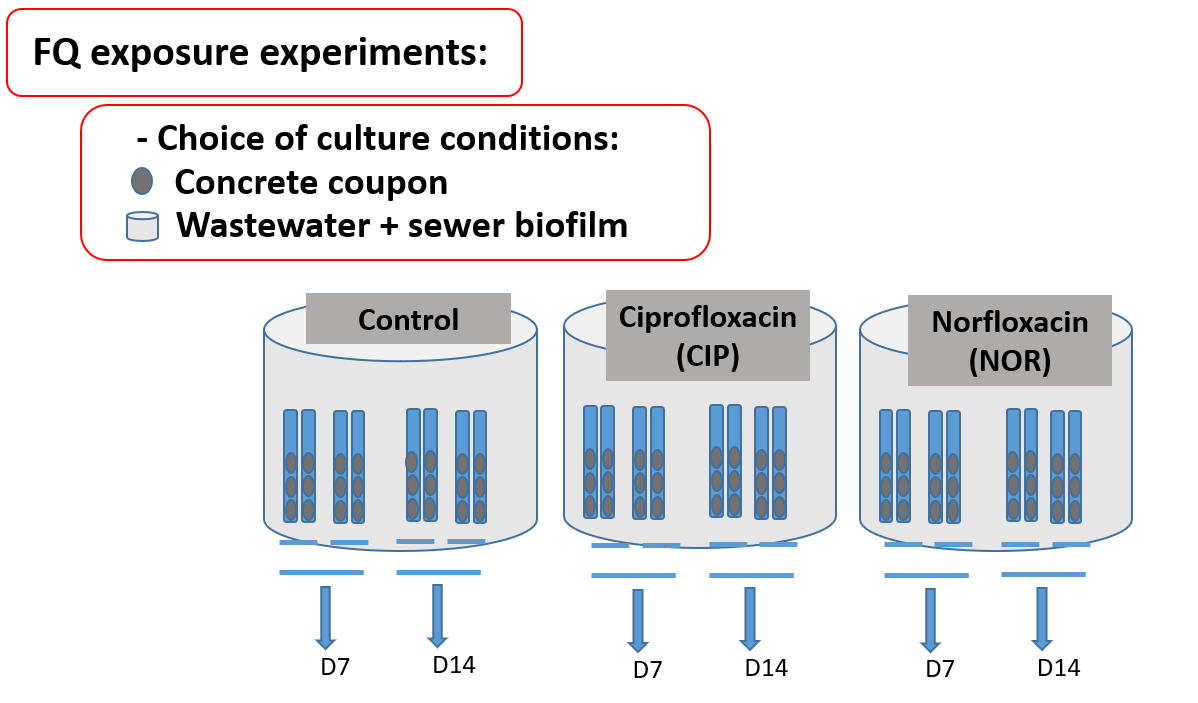


**Figure S1**. Diagram of bioreactors.

**Table S1**. Experimental design.

| **Experiment set** | **Bioreactor** | **Initial inoculum** | **Coupon material** | **FQ exposure** |
| --- | --- | --- | --- | --- |
| **1^st^** | 1 | Wastewater A | Polycarbonate and Concrete | No FQ |
|  | 2 |  |  |  |
|  | 3 | Wastewater A + sewer biofilm |  |  |
|  | 4 |  |  |  |
| **2^nd^** | 1 | Wastewater B | Polycarbonate and Concrete | No FQ |
|  | 2 |  |  |  |
|  | 3 | Wastewater B + sewer biofilm |  |  |
|  | 4 |  |  |  |
| **3^rd^** | 1 | Wastewater E + sewer biofilm | Concrete | No FQ |
|  | 2 |  |  | Ciprofloxacin (5,000 µg/L) |
|  | 3 |  |  | Norfloxacin (5,000 µg/L) |
| **4^th^** | 1 | Wastewater F + sewer biofilm | Concrete | No FQ |
|  | 2 |  |  | Ciprofloxacin (2.5 µg/L) |
|  | 3 |  |  | Norfloxacin (2.5 µg/L) |

**Table S2.** Retention times and MS parameters used for the quantification of FQ and Q.

| **Compound** | **Retention time (min)** | **Parents**  **(m/z)** | **Daughter**  **(m/z)** | **Q1 Pre Bias (V)** | **CE (v)** | **Q3**  **Pre Bias (V)** |
| --- | --- | --- | --- | --- | --- | --- |
| Oxilinic Acid | 4.76 | 262 | 244 | -15 | -16 | -27 |
|  |  | 262 | 216 | -13 | -30 | -22 |
| Flumequine | 5.58 | 262 | 244 | -18 | -17 | -26 |
|  |  | 262 | 202 | -10 | -32 | -21 |
| Norfloxacin | 3.51 | 320 | 302 | -22 | -19 | -14 |
|  |  | 320 | 231 | -24 | -39 | -24 |
| Ciprofloxacin | 3.61 | 332 | 314 | -23 | -18 | -11 |
|  |  | 332 | 231 | -23 | -37 | -23 |
| Lomefloxacin | 3.78 | 352 | 265 | -18 | -23 | -18 |
|  |  | 352 | 334 | -18 | -19 | -22 |
| Danofloxacin | 3.75 | 358 | 340 | -11 | -22 | -24 |
|  |  | 358 | 82 | -14 | -45 | -15 |
| Enrofloxacin | 3.75 | 360 | 342 | 0 | 20 | -20 |
|  |  | 360 | 316 | 0 | 20 | -20 |
| Levofloxacin | 3.53 | 362 | 261 | -11 | -28 | -18 |
|  |  | 362 | 318 | -27 | -18 | -22 |
| Marbofloxacin | 3.33 | 363 | 72 | -27 | -25 | -13 |
|  |  | 363 | 320 | -26 | -16 | -22 |
| Moxifloxacin | 4.40 | 402 | 384 | -16 | -25 | -27 |
|  |  | 402 | 358 | -30 | -19 | -26 |
|  |  |  |  |  |  |  |
| Cipro-d8(SI) | 3.60 | 340 | 322 | -13 | -22 | -22 |
|  |  | 340 | 235 | -14 | -43 | -24 |

**Table S3.** PCR primer sequences and product size for PMQR quantification by qPCR and for mutations determination using sequencing of *E. coli* *gyrA* and *parC*.

| **Gene-Direction** | **Primer sequence (5’ → 3’)** | **Product size (pb)** | **Reference** | **Accession number of target DNA for quantification** |
| --- | --- | --- | --- | --- |
| *16S rRNA*-Fw | GGGTTGCGCTCGTTGC | 60 | Zhu et al., 2013 | NR_024570.1 |
| *16S rRNA*-Rv | ATGGYTGTCGTCAGCTCGTG |  |  |  |
| *qnrA*-Fw | AGGATTTCTCACGCCAGGATT | 124 | Guo et al., 2018 | JF969163.1 |
| *qnrA*-Rv | CCGCTTTCAATGAAACTGCA |  |  |  |
| *qnrB*-Fw | GGMATHGAAATTCGCCACTG | 263 | Marti and Balcázar, 2013 | NG_057451.1 |
| *qnrB*-Rv | TTYGCBGYYCGCCAGTCGAA |  |  |  |
| *qnrC*-Fw | ATTACGGGTTGTAATTTGTCTTATG | 144 | Guillard et al., 2011 | EU917444.1 |
| *qnrC*-Rv | ATCAGAAAATGATCCCCTACT |  |  |  |
| *qnrD*-Fw | ACGACAGGAATAGCTTGGAAGG | 373 | Guo et al., 2018 | NG_050541.1 |
| *qnrD*-Rv | TCAGCCAAAGACCAATCAAACG |  |  |  |
| *qnrS*-Fw | TAAATCACACGCACGGAACT | 132 | Guo et al., 2018 | NG_048055.1 |
| *qnrS*-Rv | AACAGGGTGATATCGAAGGC |  |  |  |
| *qepA-Fw* | GCCGGTGATGCTGCTGA | 93 | Guillard et al., 2011 | NG_050459.1 |
| *qepA-Rv* | CAGRAACAGCGCSCCSA |  |  |  |
| *gyrA*-Fw | GGTACACCGTCGCGTACTTT | 311 | Johnning et al., 2015 |  |
| *gyrA*-Rv | CAACGAAATCGACCGTCTCT |  |  |  |
| *parC*-Fw | GCCTTGCGCTACATGAATTT | 287 | Johnning et al., 2015 |  |
| *parC*-Rv | ACCATCAACCAGCGGATAAC |  |  |  |

Guillard, T., Moret, H., Brasme, L., Carlier, A., Vernet-Garnier, V., Cambau, E., et al. (2011). Rapid detection of qnr and qepA plasmid-mediated quinolone resistance genes using real-time PCR. *Diagn. Microbiol. Infect. Dis.* 70, 253–259. doi: 10.1016/j.diagmicrobio.2011.01.004.

Guo, X., Yan, Z., Zhang, Y., Xu, W., Kong, D., Shan, Z., et al. (2018). Behavior of antibiotic resistance genes under extremely high-level antibiotic selection pressures in pharmaceutical wastewater treatment plants. *Sci. Total Environ.* 612, 119–128. doi: 10.1016/j.scitotenv.2017.08.229.

Johnning, A., Kristiansson, E., Angelin, M., Marathe, N., Shouche, Y. S., Johansson, A., et al. (2015). Quinolone resistance mutations in the faecal microbiota of Swedish travellers to India. *BMC Microbiol.* 15, 235. doi: 10.1186/s12866-015-0574-6.

Marti, E., and Balcázar, J. L. (2013). Real-Time PCR Assays for Quantification of qnr Genes in Environmental Water Samples and Chicken Feces. *Appl. Environ. Microbiol.* 79, 1743–1745. doi: 10.1128/AEM.03409-12.

Zhu, Y.-G., Johnson, T. A., Su, J.-Q., Qiao, M., Guo, G.-X., Stedtfeld, R. D., et al. (2013). Diverse and abundant antibiotic resistance genes in Chinese swine farms. *Proc. Natl. Acad. Sci.* 110, 3435–3440. doi: 10.1073/pnas.1222743110.


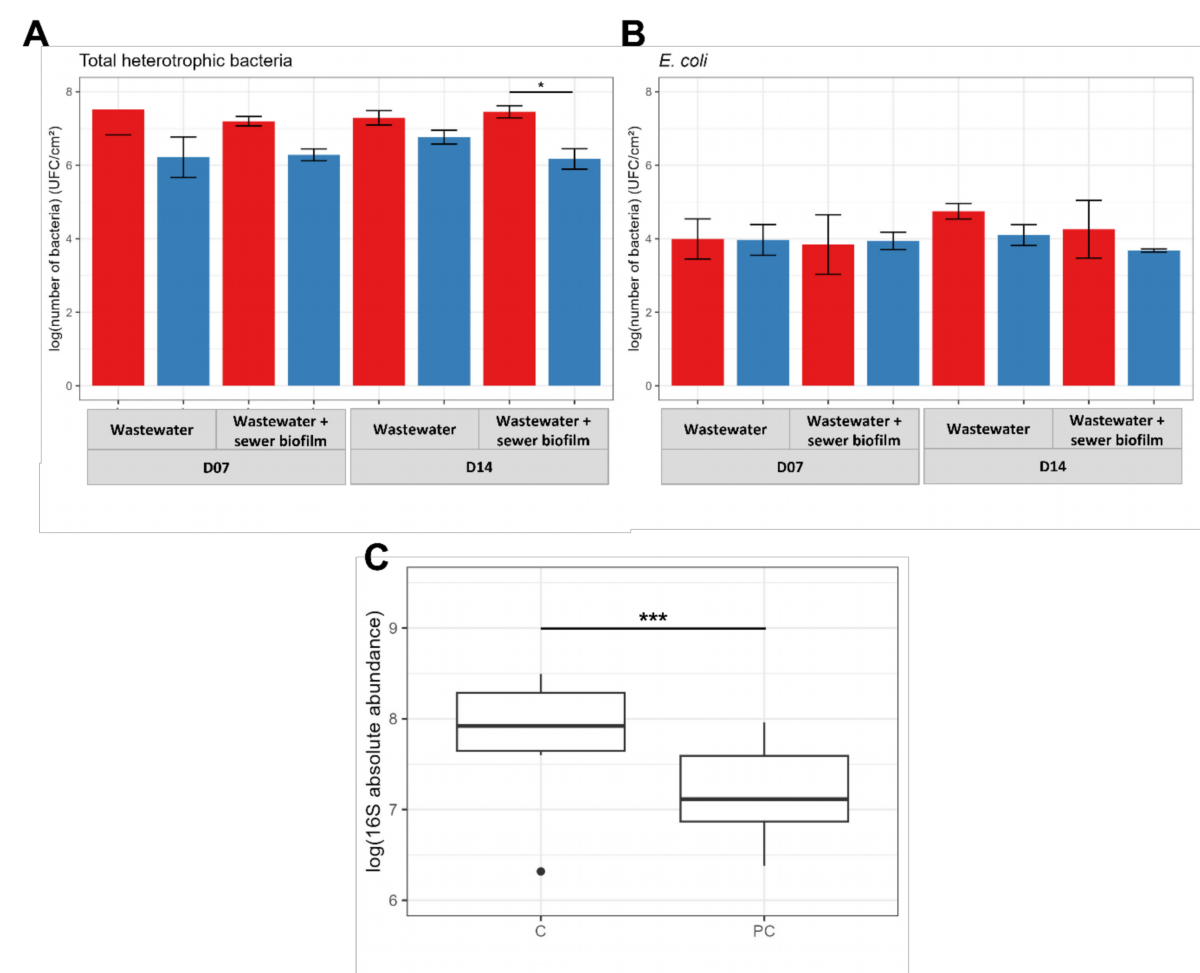


**Fig S2.** Heterotrophic bacteria (**A**) and *E. coli* (**B**) counts in *in vitro* biofilms at D07 and D14. Means of the 1^st^ and 2^nd^ experiment. C, concrete; PC, polycarbonate. Samples were grouped by coupon material (concrete, red and polycarbonate, blue) and initial inoculation condition (wastewater without or with sewer biofilm). (**C**) Absolute abundance of the log of the quantity of 16S rRNA gene per sampling area for *in vitro* biofilms. Samples were grouped by coupon material (concrete, C; polycarbonate, PC). Statistical differences were assessed with Kruskal-Wallis test followed by Dunn post-hoc test for A and B, and Wilcoxon signed-rank test for C (*: p < 0.05; ***: p < 0.001).


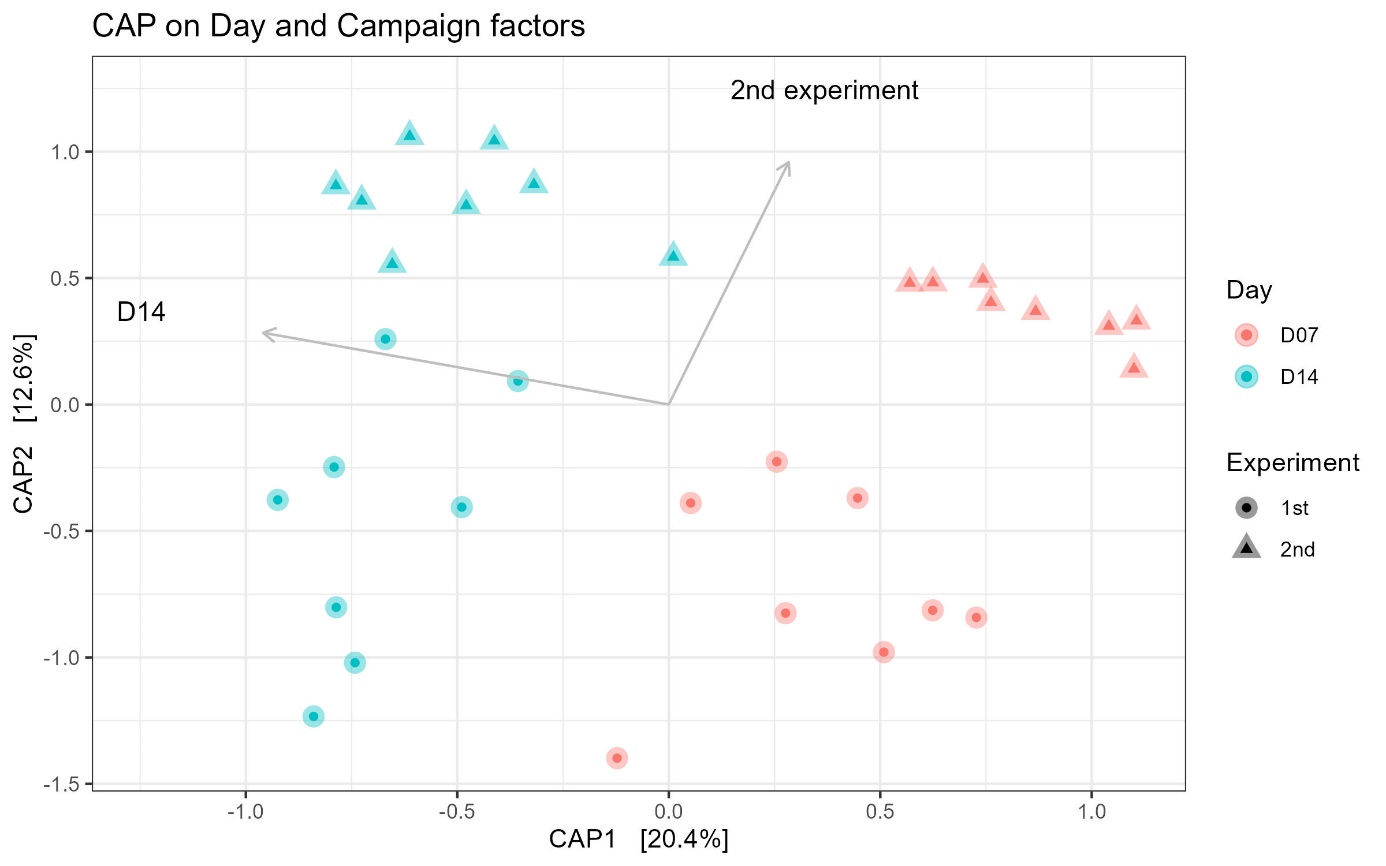


**Fig S3.** Visualization by CAP (Constrained Analysis of Principal coordinates) ordination method of the β-diversity analysis carried out using the Bray-Curtis dissimilarity index, constrained by the day (D07 and D14) and the experiment (1^st^ and 2^nd^).


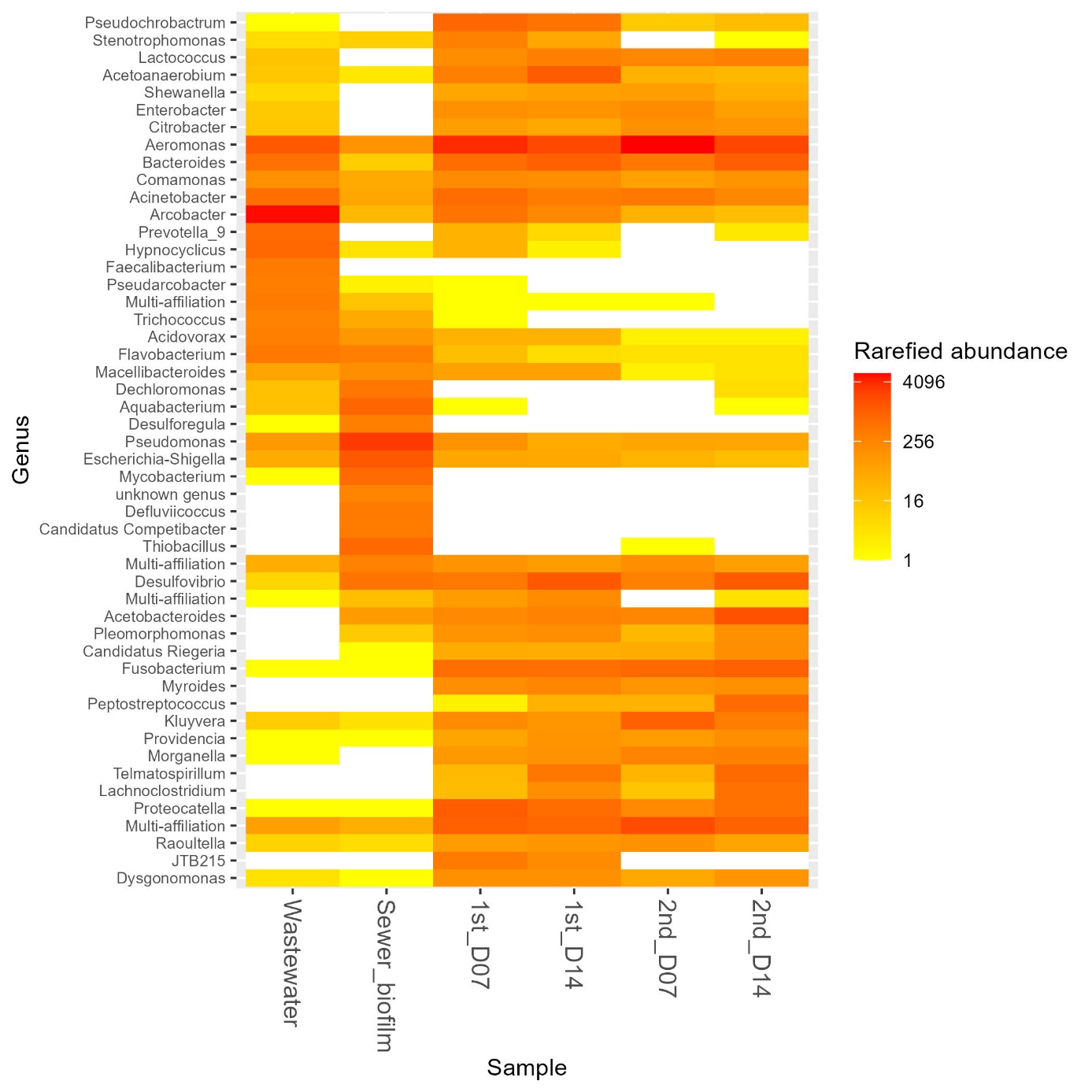


**Fig S4.** Heatmap of the 50 most abundant genera after rarefaction. Abundances were averaged for each sample type.

**Table S4.** Relative abundance of mutations in *E. coli* *gyrA* at codons S83 and D87 in field samples and in *in vitro* biofilms at D14 not exposed to FQs depending on the material of the coupons (polycarbonate and concrete) and the addition or not of sewer biofilm at the initial inoculation of bioreactors, and in *in vitro* biofilms at D14 exposed to FQs (CIP, ciprofloxacin and NOR, norfloxacin) at low and high exposure.

| **Relative abundance of mutation in *E. coli* (%)** | | | | | | | | |
| --- | --- | --- | --- | --- | --- | --- | --- | --- |
|  | **Field samples** | | ***In vitro* biofilms without FQs** | | | | | |
|  | Wastewater | Sewer biofilm | Polycarbonate | Polycarbonate | Concrete | Concrete | Mean (SD) | |
|  |  |  | Without sewer biofilm | With sewer biofilm | Without sewer biofilm | With sewer biofilm |  |  |
| **S83L** | 14.3 | 0.9 | 18.1 | 11.9 | 21.3 | 12.7 | 16.0 ± 4.5 | |
| **S83G** | 5.4 | 6.7 | 2.7 | 4.1 | 2.5 | 3.9 | 3.3 ± 0.8 | |
| **S83A** | 1.2 | 0.4 | 2.7 | 0.6 | 4.6 | 0.8 | 2.2 ± 1.9 | |
| **D87N** | 7.3 | 1.5 | 13.7 | 7.0 | 18.8 | 8.5 | 12.0 ± 5.4 | |
| **D87L** | 4.9 | 6.2 | 2.1 | 3.7 | 2.0 | 3.3 | 2.8 ± 0.9 | |
| **D87E** | 0.2 | 0.3 | 3.0 | 0.3 | 1.7 | 0.3 | 1.3 ± 1.3 | |
|  |  |  | ***In vitro* biofilms with FQs** | | | | | |
|  |  |  | Control | | CIP | | NOR | |
|  |  |  |  |  | Low Exposure | High Exposure | Low Exposure | High Exposure |
| **S83L** |  |  | 8.8 | 9.6 | 57.2 | 82.5 | 18.2 | 85.6 |
| **S83G** |  |  | 2.9 | 4.9 | 4.3 | 6.8 | 2.8 | 4.6 |
| **S83A** |  |  | 0.1 | 0.2 | 0.5 | 1.1 | 0.3 | 1.1 |
| **D87N** |  |  | 7.2 | 5.5 | 6.5 | 87.9 | 7.0 | 90.5 |
| **D87L** |  |  | 2.2 | 4.5 | 3.5 | 6.4 | 2.3 | 4.4 |
| **D87E** |  |  | 2.8 | 0.3 | 1.5 | 0.1 | 7.7 | 0.2 |

**Table S5.** Relative abundance of mutations in *E. coli* *parC* at codons S80 and E84 in field samples and in *in vitro* biofilms at D14 not exposed to FQs depending on the material of the coupons (polycarbonate and concrete) and the addition or not of sewer biofilm at the initial inoculation of bioreactors , and in *in vitro* biofilms at D14 exposed to FQs (CIP, ciprofloxacin and NOR, norfloxacin) at low and high exposure.

| **Relative abundance of mutation in *E. coli* (%)** | | | | | | | | |
| --- | --- | --- | --- | --- | --- | --- | --- | --- |
|  | **Field samples** | | ***In vitro* biofilms without FQs** | | | | | |
|  | Wastewater | Sewer biofilm | Polycarbonate | Polycarbonate | Concrete | Concrete | Mean (SD) | |
|  |  |  | Without sewer biofilm | With sewer biofilm | Without sewer biofilm | With sewer biofilm |  |  |
| **S80I** | 8.7 | 8.3 | 18.5 | 9.1 | 18.9 | 10.2 | 14.2 ± 5.2 | |
| **S80V** | 4.6 | 4.4 | 4.7 | 4.5 | 4.7 | 4.0 | 4.5 ± 0.3 | |
| **S80G** | 1.1 | 1.1 | 1.2 | 1.1 | 1.2 | 1.1 | 1.2 ± 0.1 | |
| **E84V** | 1.8 | 0.4 | 1.0 | 0.5 | 0.7 | 0.4 | 0.7 ± 0.3 | |
| **E84R** | 1.2 | 1.3 | 1.2 | 1.1 | 1.3 | 1.0 | 1.2 ± 0.1 | |
| **E84K** | 0.9 | 0.9 | 1.0 | 0.9 | 1.0 | 0.8 | 0.9 ± 0.1 | |
|  |  |  | ***In vitro* biofilms with FQs** | | | | | |
|  |  |  | Control | | CIP | | NOR | |
|  |  |  |  |  | Low Exposure | High Exposure | Low Exposure | High Exposure |
| **S80I** |  |  | 9.0 | 6.0 | 17.5 | **75.5** | 2.4 | **75.8** |
| **S80V** |  |  | 3.2 | 3.7 | 4.3 | 5.7 | 4.2 | 5.7 |
| **S80G** |  |  | 1.1 | 1.2 | 0.9 | 1.0 | 1.2 | 0.9 |
| **E84V** |  |  | 1.3 | 0.5 | 0.4 | 4.4 | 0.5 | 4.5 |
| **E84R** |  |  | 0.7 | 1.0 | 1.2 | 1.4 | 1.2 | 1.5 |
| **E84K** |  |  | 1.0 | 1.0 | 3.6 | 1.0 | 0.8 | 1.0 |

**Table S6.** Differential abundance of ARGs grouping according to resistance to antibiotic classes. ARGs that had an average ≥ 10 readings in at least one of the sample categories (sewer biofilm, wastewater or *in vitro* biofilms) were included. ^a^, differential abundance of ARG counts were represented with the score scale Log2 fold change. Boxed results represented Log2FC ≤ -1.5 or ≥ 1.5 and p-adjusted value < 0.05. ^b^, the counts of the ARGs were analysed with Poisson regression model and a FDR correction for multiple testing (p < 0.05 (*), p < 0.01 (**), p < 0.001 (***)). MS, macrolide_streptogramin; MLS, macrolide_lincosamide_streptogramin; LSOP, lincosamide_streptogramin_oxazolidinone_phenicol; SP, streptogramin_pleuromutilin; PLS, pleuromutilin_lincosamide_streptogramin; OP, oxazolidinone_phenicol. MS, erm (RNA methyl tranferase, target alteration); ML, tlcR (ABC-F subfamily protein, target protection) and RmlA(II) (non-erm 23S rib methyltransferase (target alteration); LSOP, cfr-like 23S RNA methyltransferase (target alteration); SP, vga-type plasmid-borne ABC-F proteins; PLS, Isa-type-ABC-F prot -ribosome protection); OP, optrA as ABC-F subfamily (ribosome protection).


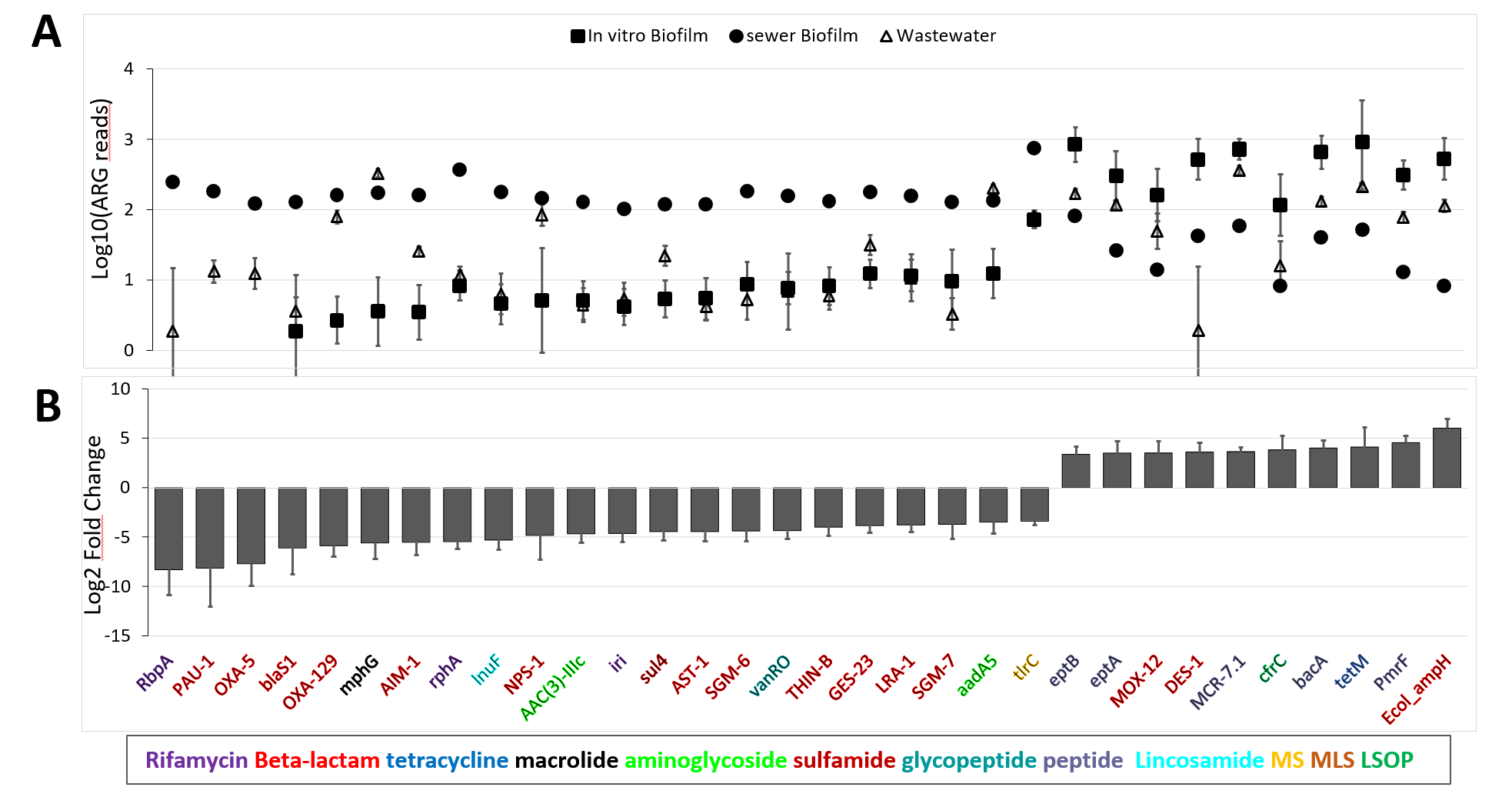


**Figure S5.** Differential abundance of ARGs. (**A**) Abundance of ARG reads in sewer biofilm, wastewater and *in vitro* biofilms and (**B**) Changes in ARG abundances. Bars represent Log2 fold change (*in vitro* biofilm *vs* sewer biofilm) ± standard deviation. Only ARGs with read counts ≥ 100 in *in vitro* biofilms or in sewer biofilm and -3.32<Log2FC<3.32 were represented in the graph.

**Table S7.** Differential abundance of EPGs grouping according to resistance to antibiotic classes. EPGs that had an average ≥ 10 readings in at least one of the sample categories (sewer biofilm, wastewater or *in vitro* biofilms) were included. ^a^, differential abundance of EPGs was represented with the score scale Log2 fold change. Boxed results represented Log2FC ≤-1.5 or ≥1.5 and p-value <0.05. ^b^, the counts of the EPGs were analysed with Poisson regression model and a FDR correction for multiple testing (p < 0.05 (*), p < 0.01 (**), p < 0.001 (***)).

**Figure S6:** Differential abundance of EPGs. (A) Abundance of EPG reads in sewer biofilm, in wastewater and *in vitro* biofilms and (B) changes in EPG abundances. Bars represent Log2 fold change (*in vitro* biofilms *vs* sewer biofilm) ± standard deviation. Only EPGs with read counts ≥ 100 in *in vitro* biofilms or in sewer biofilm and -3.32<Log2FC<3.32 were represented in the graph.


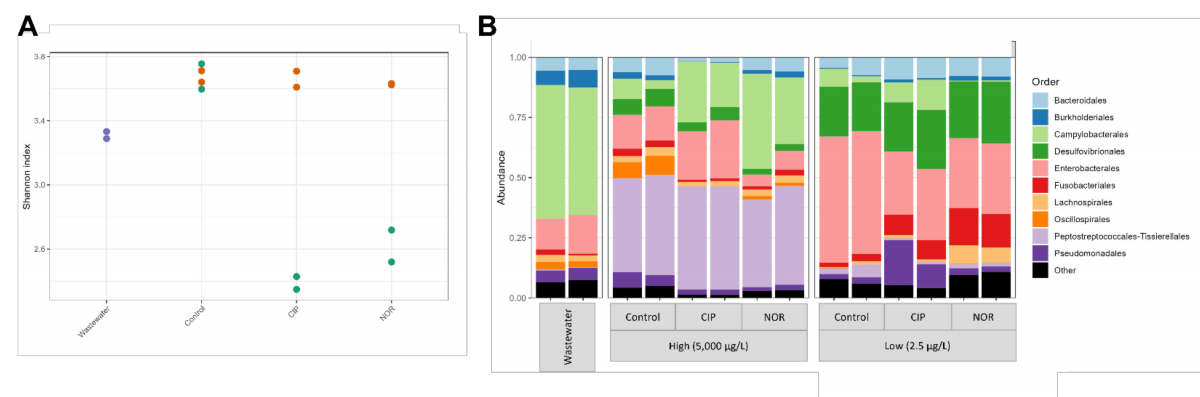


**Figure S7.** (**A**) Alpha diversity (Shannon index) of wastewater samples and *in vitro* biofilms as a function of the exposure (Control, without FQ; CIP, ciprofloxacin and NOR, norfloxacin) and the concentration (high, 5,000 µg/L, green and low, 2.5 µg/L, red). (**B**) Bar chart representing the relative abundance of the 10 majority orders for *in vitro* biofilms non-exposed (Control) and exposed (High and Low) to two FQs (CIP and NOR).


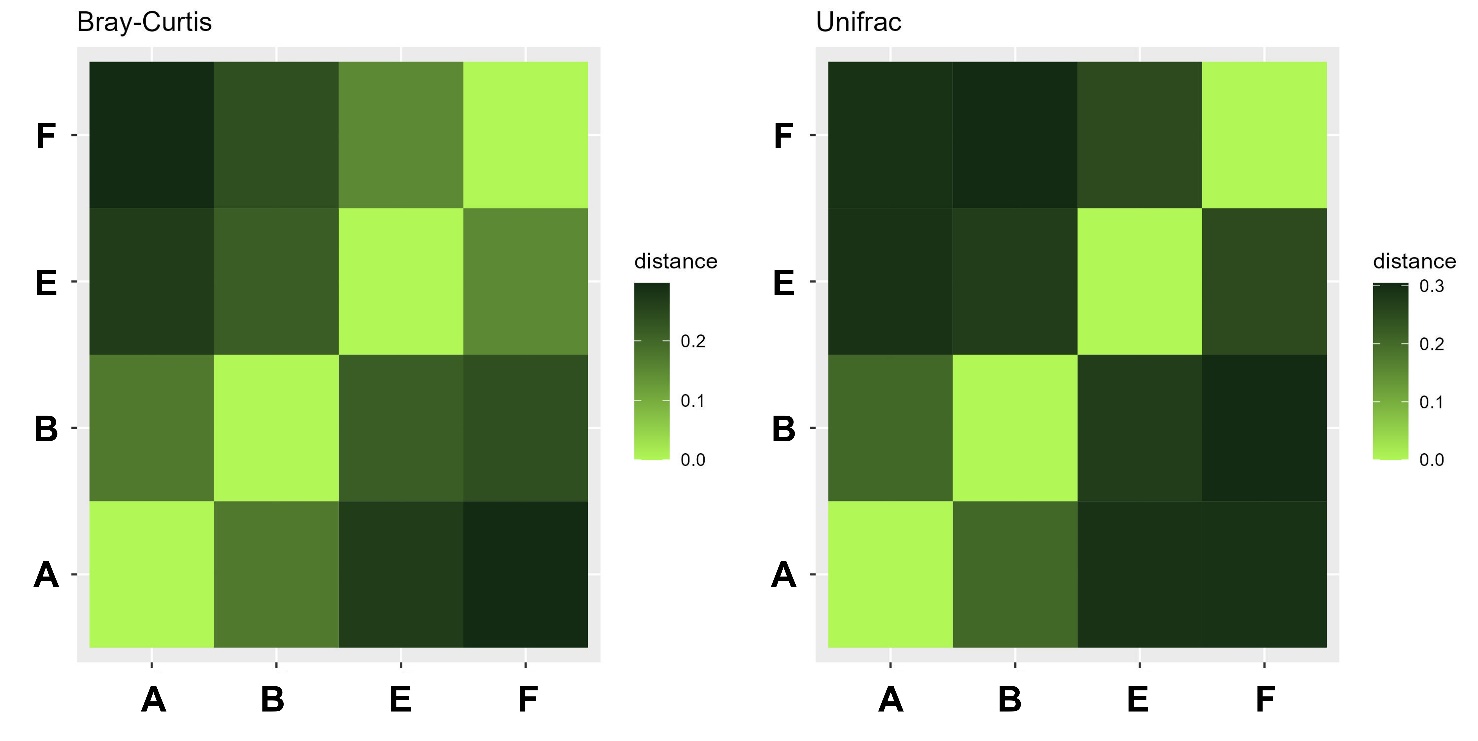


**Figure S8.** Bray-Curtis and Unifrac diversity index measured between four wastewater samples (A, B, E and F).
